# Capsid stabilization reprograms the nuclear fate of the HIV genome

**DOI:** 10.1126/sciadv.aef9469

**Published:** 2026-08-07

**Authors:** Thierry Mourer, Mohamad Harastani, Celine Cuche, Jose Ignacio Perez-Lopez, Salvatore Tornambe, Stephane Tachon, Guillaume Corre, Maria Julia Maristany, Jean-Yves Tinevez, Anastasia Gazi, Valentina Poletti, Rosana Collepardo-Guevara, Anna Sartori-Rupp, Francesca Di Nunzio

**Affiliations:** ^1^ Virology Department, Institut Pasteur, Université Paris Cité, Paris, France.; ^2^ Image Analysis Hub (IAH), Institut Pasteur, Université Paris Cité, Paris, France.; ^3^ Yusuf Hamied Department of Chemistry, University of Cambridge, Cambridge, UK.; ^4^ NanoImaging Core Facility (NCF), Institut Pasteur, Université Paris Cité, Paris, France.; ^5^ Genethon, Evry, France.; ^6^ Université Paris-Saclay, University of Evry, Inserm, Généthon, Integrare Research Unit UMR_S951, Evry-Courcouronnes, France.; ^7^ Ultrastructural BioImaging Facility (UBI), Institut Pasteur, Université Paris Cité, Paris France.; ^8^ Division of Pediatric Hematology, Oncology and Stem Cell Transplant, Department of Women’s and Children’s Health, University of Padua, Padua, Italy.; ^9^ Research Area of Pediatric Hematology, Oncology and Hematopoietic Cell and Gene Therapy, Pediatric Research Institute Fondazione Città Della Speranza, Padua, Italy.; ^10^ Department of Genetics, University of Cambridge, Cambridge, UK.

## Abstract

Capsid stabilization, induced by low-nanomolar concentrations of Lenacapavir (LEN)—a first-in-class capsid inhibitor—impedes HIV-1 replication in macrophages without disrupting reverse transcription or nuclear import. In LEN-treated cells, ultrastructural analyses reveal preserved conical capsids persisting within nuclear CPSF6-enriched puncta—HIV-1–induced membraneless organelles (HIV-1–MLOs)—which remain spatially segregated from canonical integration hubs near nuclear speckles (NSs). Rather than fusing with NSs, HIV-1–MLOs are rerouted to chromatin-associated promyelocytic leukemia nuclear bodies (PML-NBs), where molecular exchange occurs without condensate fusion. This nuclear redirection correlates with the sequestration of the viral genome within stabilized capsids, reducing the pool of viral DNA available for integration and redirecting the few accessible genomes to atypical integration sites near PML-NBs. These findings uncover a previously unrecognized mechanism of LEN action, whereby capsid stabilization reprograms the nuclear fate of the HIV-1 genome in macrophages, limiting integration and redirecting viral DNA toward more repressive nuclear environments, likely shaping the transcriptional potential of newly integrated viral DNA.

## Teaser

Ultrastructural analysis reveals that LEN stabilizes capsids in nuclei, reprogramming HIV-1 trafficking to PML-NBs.

## INTRODUCTION

HIV type 1 (HIV-1) is the etiological agent behind one of the most devastating pandemics in the history and remains among the most studied human pathogens for over four decades (*1*). Advances from basic virology enabled the rational design of antiretroviral drugs, transforming HIV-1 infection from a fatal disease into a manageable chronic condition (*2*). A past landmark in HIV-1 treatments was the approval of saquinavir in 1995, the antiviral developed using structure-based drug design (*3*). Nevertheless, saquinavir and other antiretroviral drugs, used in combination as part of antiretroviral therapy, require daily administration and do not provide a cure (*4*). The absence of a definitive cure against HIV-1 may stem from an incomplete understanding of its life cycle. Recent studies have revisited early models of HIV-1 nuclear entry and uncoating. The viral core is now recognized as an active participant in infection, guiding cytoplasmic trafficking (*5*, *6*), nuclear import (*7*–*16*), and post-nuclear events (*17*). This overturns the earlier assumption that capsid disassembles immediately after fusion with the cellular membrane (*18*). Imaging and biochemical studies instead demonstrate that intact capsids persist until nuclear entry (*15*–*17*, *19*, *20*), thereby reframing the capsid as a druggable antiviral target. These insights contributed to the development of the first-in-class capsid inhibitor Lenacapavir (LEN), highlighted as “Breakthrough of the Year 2024” by *Science* magazine (*21*, *22*).

LEN was initially approved for patients with multidrug-resistant HIV-1 (*23*) and more recently for use as long-acting preexposure prophylaxis (*24*). In clinical settings, LEN injection reaches a maximal peak plasma concentration of ∼100 nM and then stabilizes at ∼26 nM during maintenance (*25*, *26*). Although its tissue distribution in humans remains incompletely characterized, available animal studies indicate that LEN distributes across multiple tissues, with levels that may vary depending on the organ (*27*). While clinical studies indicate that LEN achieves sustained plasma concentrations in the low nanomolar range, the pharmacologically active free drug fraction is substantially lower due to extensive protein binding (*27*), and its effective intracellular concentration within tissue-resident macrophages remains unknown. Given these uncertainties and known cell-type differences in drug handling, our use of low-nanomolar LEN concentrations provides a controlled framework to investigate its mechanism of action in macrophages without assuming direct equivalence to systemic exposure. Some mechanistic studies to date have applied LEN on isolated cell-free core structures or used coarse-grained simulations (*28*–*31*), during viral production by plasmid transfection (*32*), or in infected cells with LEN applied postinfection (*31*, *33*, *34*). However, these approaches were unable to detail features of viral cores in situ directly within the nucleus. In this study, we aimed to more closely reproduce physiological acute infection conditions while examining LEN activity at low-nanomolar concentrations. We administered LEN at the time of infection rather than postinfection, as viral cores do not persist alongside the viral genome for extended periods. This approach enables an accurate investigation of LEN’s mechanism of action during early stages of infection, including nuclear entry, within a biologically relevant concentration range. Given that HIV-1 replication during chronic infection primarily occurs within lymphoid tissues and that macrophages might contribute to viral persistence depending on their tissue residence and degree of viral suppression in people living with HIV on antiretroviral therapy (*35*–*37*). In addition, these cells are a good model to explore the role of LEN on HIV-1–induced membraneless organelles (HIV-1–MLOs) that remain unexplored. During a canonical infection, the host nuclear factor, cleavage and polyadenylation specificity factor 6 (CPSF6), through its phenylalanine-glycine (FG) domain, engages the viral core upon nuclear entry and simultaneously participates in the formation of HIV-1–MLOs. HIV-1–MLOs are readily detectable by confocal microscopy at high multiplicity of infection (MOI) (>1). Notably, the amount of virus transmitted during cell-to-cell contact is relatively high, closely mirroring the viral loads achieved under high MOI conditions in vitro (*38*) and those observed during physiological infection in animal models (*17*). These MLOs rapidly fuse with a subset of nuclear speckles (NSs) (*39*, *40*). HIV-1 exploits phase separation mechanisms to generate these nuclear MLOs, which function as sites for canonical viral reverse transcription, protection from innate immune sensing, and targeting of the viral genome to speckle-associated domains (SPADs), thereby promoting efficient viral replication (*17*, *41*, *42*).

To explore how capsid-targeting antivirals act on viral cores retained in HIV-1–MLOs and how these organelles are consequentially affected in macrophages, we examined the effects of LEN using a low-nanomolar concentration within the range of reported protein-adjusted antiviral potency (paEC_95_ ≈ 3.87 ng/ml, ∼4 nM), as a relevant benchmark for mechanistic investigations (*43*, *44*). We show that 5 nM LEN suppresses HIV-1 replication without disrupting reverse transcription or affecting nuclear import. Instead, its effect emerges during post-nuclear stages of the viral life cycle. Leveraging technologies such as cryo–electron tomography, coarse-grained molecular simulations, and advanced fluorescence imaging, we explored how capsid stabilization influences the spatial organization and functional fate of the HIV-1 genome within the nucleus. We achieved an unprecedented structural reconstruction of nuclear HIV-1 cores in situ, capturing a high frequency of preserved conical capsids within infected macrophage nuclei. These observations reveal that LEN-stabilized cores remain compartmentalized within HIV-1–MLOs, which contain both CPSF6 and viral cores (*17*, *39*), while being spatially segregated from host nuclear condensates such as NSs. This redistribution brings LEN-stabilized cores within HIV-1–MLOs into closer proximity with noncanonical HIV nuclear environments, such as promyelocytic leukemia nuclear bodies (PML-NBs), demonstrating that capsid stabilization reprograms the nuclear fate of the HIV-1 genome and offers a previously unidentified framework for limiting reservoir persistence.

## RESULTS

### LEN inhibits HIV-1 infection by targeting post-nuclear entry events

In this study, we investigate the impact of LEN on HIV-1 infection in macrophages, focusing on LEN impact. To assess nuclear entry, we analyze the formation of HIV-1–MLOs by treating cells with escalating doses of LEN (0.5 nM, 5 nM, 50 nM, or 5 μM) from the onset of infection using a single-round HIV-1 carrying integrase fused to an hemagglutinin (HA) tag (*15*, *17*, *39*, *45*). We have also used PF74, a capsid (CA) inhibitor, at low dose (1.25 μM) as it has been described to block nuclear import (*15*, *46*, *47*). Without LEN treatment, CPSF6 immunolabeling reveals HIV-1–MLOs formation (82 CPSF6 puncta identified in 100 cells) (Fig. 1, A and B). LEN at 0.5 or 5 nM modestly reduced MLO assembly (71 or 68 CPSF6 puncta per 100 cells) (Fig. 1, A and B, and fig. S1). In contrast, LEN at 50 nM or 5 μM completely blocks nuclear import, similar to PF74, as evidenced by the absence of MLO formation. This stands in stark contrast to treatment with nevirapine (NVP), a reverse transcriptase inhibitor, or raltegravir (RAL), an integrase inhibitor, where the number of condensates remains comparable to that observed in DMSO-treated controls. (Fig. 1, A and B, and fig. S1).

**Fig. 1.**
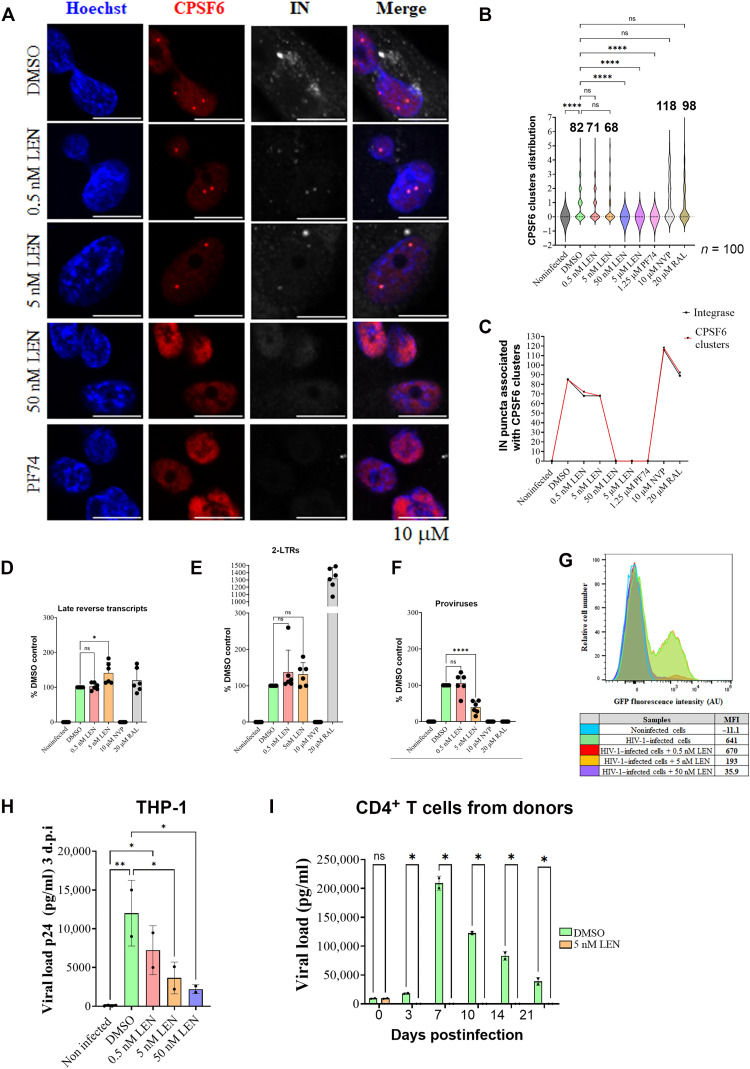
LEN (5 nM) disrupts HIV-1 replication cycle through a mechanism that is independent of both reverse transcription and nuclear entry. (**A**) THP-1 cells were differentiated with PMA and infected with VSV-G/HIV-1 ∆Env (IN_HA_) 72 hours (MOI = 20) in the absence or presence of inhibitors. Immunofluorescence shows CPSF6 (red), the IN (white), and nuclei (blue). Scale bars, 10 μm. (**B**) Violin plot showing the number of CPSF6 puncta distribution in the population of each condition. Statistical test: Ordinary One-Way ANOVA test (****P < 0.0001) from two independent experiments on 100 randomly selected cells. (**C**) Graph shows the colocalization between CPSF6 clusters (red line) and IN (black line). (**D**) THP-1 cells were infected for 72 hours and were treated or not with the following drugs: 0.5 nM LEN (red), 5 nM LEN (orange), 10 μM NEV (pink), and 20 μM RAL (gray). Histograms show LRTs (ns = 0.99; *P = 0.01). (**E**) 2-LTRs (ns = 0.92 to 0.96). (**F**) Proviruses (****P = 0.00000015; ns = 0.99). Statistical test: ordinary one-way ANOVA. (**G**) FACS analysis of THP-1 cells infected for 72 hours with HIV-1 ∆Env VSV-G carrying the GFP reporter gene: blue, untreated; green, infected; red, infected + 0.5 nM LEN; orange, infected + 5 nM LEN; purple: infected + 50 nM LEN. Table shows the mean fluorescence intensities (MFIs) from three independent experiments. (**H**) At 3 days postinfection (d.p.i), HIV-1 (NL4-3-AD8) replication was assessed in the presence of LEN (0.5 nM, red; 5 nM, orange; and 50 nM, purple) and in the absence of LEN (green bar). p24 level are shown as the means ± SD. Statistical test: ordinary one-way ANOVA [**P = 0.0023; *P = 0.0364 (top), 0.034 (middle), and 0.015 (bottom)]. Data are representative of two independent experiments. (**I**) NL4.3-AD8 replication at MOI = 0.5 was monitored for 21 days in primary CD4^+^ T cells by p24 ELISA. Statistical test is from two healthy donors: unpaired *t* test [not significant (ns) > 0.99; *P < 0.05].

Colocalization studies confirm that CPSF6 puncta represent bona fide HIV-1–MLOs also in presence of LEN, as nearly all puncta contain HA-tagged integrase (Fig. 1C). We next evaluate the effect of LEN on the reverse transcription. Building on prior evidence that reverse transcription takes place within HIV-1–MLOs (*17*, *39*), we quantify late reverse transcripts (LRTs) using real-time polymerase chain reaction (PCR). LEN at low concentrations (0.5 and 5 nM) does not significantly affect reverse transcription efficiency, in line with previous studies (Fig. 1D) (*28*).

Nuclear import of viral cores was also assessed by quantification of 2–long terminal repeat (LTR) circles–episomal DNA forms marking nuclear localization of reverse-transcribed genomes. As expected, no 2-LTRs were detected in uninfected or NVP-treated cells, while there was an increase in presence of RAL. In contrast, infected cells showed similar levels of 2-LTR circles as those observed with 0.5 and 5 nM LEN, respectively (Fig. 1E). These results indicate that LEN at these concentrations preserves reverse transcription and nuclear import. The evaluation of proviral integration by Alu-PCR revealed an approximately fourfold decrease in the number of integrated proviruses at 5 nM LEN (Fig. 1F). To evaluate the functional impact of LEN on infection, we used a single-round vesicular stomatitis virus glycoprotein G (VSV-G) pseudotyped HIV-1ΔEnv–green fluorescent protein (GFP) virus in THP-1 macrophage-like cells. LEN at 0.5 nM had no effect on infectivity [mean fluorescence intensity (MFI) = 670] compared to untreated cells (MFI = 641), whereas 5 and 50 nM reduced infectivity to MFI = 193 (∼3.4-fold) and 35.9 (∼18-fold), respectively (Fig. 1G). Thus, LEN at 5 nM significantly suppresses HIV-1 infection despite no relevant effect on reverse transcription or nuclear import. To model clinical efficacy, we infected THP-1 and primary CD4^+^ T cells with replication-competent HIV-1. In THP-1 cells, p24 levels decreased from 12,500 to 4000 pg/ml at 5 nM and to 2500 pg/ml at 50 nM LEN by day 3 postinfection (Fig. 1H). In CD4^+^ T cells, 5 nM LEN completely blocks detectable replication over 21 days (Fig. 1I). In summary, our data demonstrate that LEN at 5 nM inhibits HIV-1 replication by acting on post-nuclear entry events, without major disruption of nuclear import or reverse transcription.

### LEN preserves HIV-1–MLO, shielding vDNA from innate immunity, unlike higher concentrations

We next assess the fate and spatial distribution of viral DNA (vDNA) within the nuclei of differentiated THP-1 cells. To detect newly synthesized vDNA, we added EdU (5-ethynyl-2′-deoxyuridine) during infection. Although EdU is not inherently selective for vDNA, its incorporation is restricted to vDNA synthesis in this context because THP-1 macrophage-like cells are non-dividing. As a result, EdU specifically labels newly synthesized vDNA without marking host chromatin (Fig. 2A and fig. S2). The quantification of EdU foci per nucleus colocalized with CPSF6 puncta reveals comparable numbers in the presence and absence of 5 nM LEN (∼2.7 versus ∼2.4, respectively) (Fig. 2B). In contrast, EdU foci located outside CPSF6 puncta are markedly reduced in the presence of 5 nM LEN (∼3.4) compared with untreated cells (∼7.2) (Fig. 2C). These findings suggest that LEN promotes sequestration of vDNA within CPSF6 puncta.

**Fig. 2.**
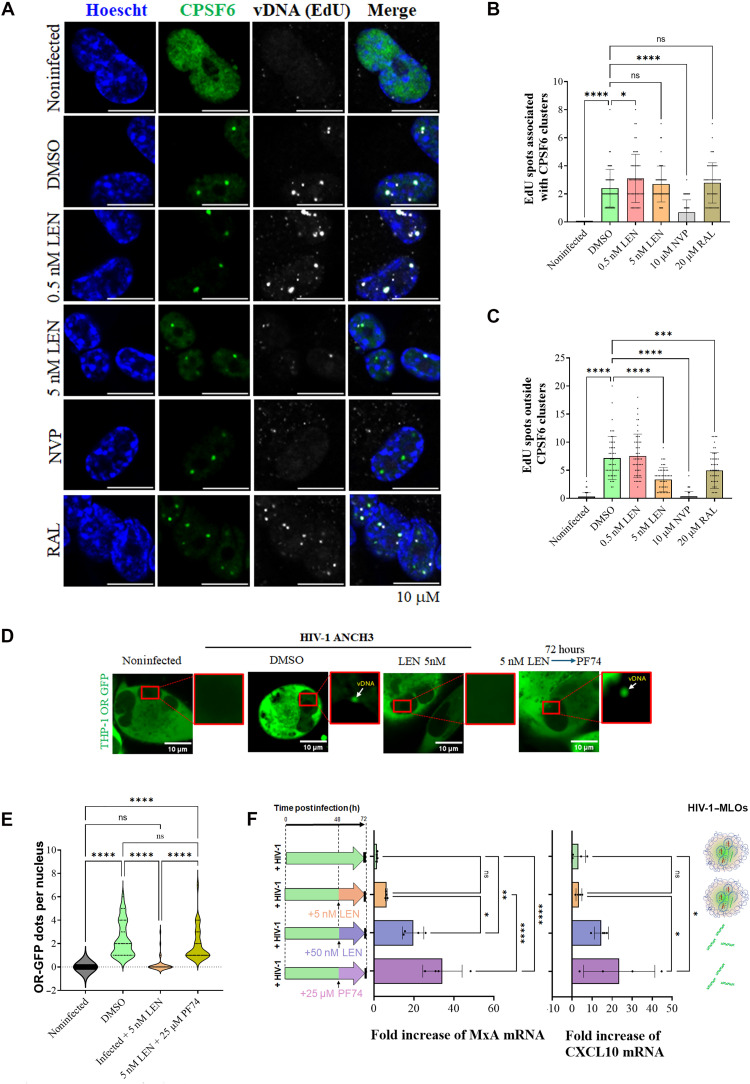
LEN (5 nM) retains vDNA in CPSF6 puncta, preventing immune sensing. (**A**) Confocal images showing nuclei (blue), CPSF6 (green), and the vDNA (EdU, white) after 72 hours of infection with VSV-G/HIV-1 ΔEnv (MOI = 20) in presence of LEN, NVP, and RAL (*n* = 50 cells). (**B**) Histogram presenting the quantification of EdU spots that associate with CPSF6 puncta. (**C**) Histogram showing the number of EdU spots outside of CPSF6 puncta. The results are the means ± SD per cell. Statistical test: ordinary one-way ANOVA (****P < 0.0001) from two independent experiments. (**D**) THP-1 cells stably expressing OR-GFP were infected with HIV-1 ANCH3 (MOI = 20) for 3 days in the presence or in the absence of LEN (5 nM). Seventy-two hours after infection, some samples were treated with PF74 (25 μM). Z-stack slices showing the double stranded vDNA (OR-GFP dots) in the green channel. Specific nuclear areas are highlighted with red squares. (**E**) Violin plots showing the whole distribution of OR-GFP molecules per nucleus in each population. Statistical analysis was performed with ordinary one-way ANOVA test (****P < 0.0001, ***P = 0.0006, and **P = 0.0047). The experiment was performed independently three times. (**F**) After 48 hours of infection (MOI = 10) with HIV-1 ∆Env pseudotyped with VSV-G, cells were treated with PF74 (25 μM) and LEN (5 and 50 nM) or left untreated for an additional 24 hours. The expression of interferon-stimulated-genes (MxA, CXCL10) was monitored by RT-qPCR. The cartoons on the right, created in BioRender. F. Di Nunzio (2026) https://BioRender.com/j0df6qa, illustrate the state of HIV-1–MLOs under the different experimental conditions described. Histograms show the fold increase of MxA and CXCL10 mRNA levels compared to untreated cells ± SD. The statistical analysis was done using the ordinary one-way ANOVA test. For MxA ****P < 0.0001, **P < 0.005, and *P < 0.05. For CXCL10, *P < 0.05. Data are representative of two independent experiments. Scale bars, 10 μm.

To further test this hypothesis, we use the HIV-1 ANCHOR system (*15*, *39*), in which the final product of the reverse transcription, the double-stranded (ds) vDNA carrying the ANCH3 sequence becomes fluorescent upon binding to OR-GFP proteins (*48*). Notably, in LEN-treated cells (5 nM), vDNA is not visible, suggesting that steric hindrance within HIV-1–MLOs prevents recognition of the ANCH3 tag by OR-GFP. We reasoned that dismantling MLOs would unmask vDNA (*17*). Accordingly, we treated infected cells at 3 days postinfection with a high dose of PF74, which disrupts HIV-1–MLOs. At high doses, PF74 competes with CPSF6 for binding to the hydrophobic pocket of the viral core (*49*), thereby disrupting HIV-1–MLOs (*17*). As predicted, vDNA signals become visible after PF74 treatment, reflecting OR-GFP binding to the ANCH3 tag sequence (Fig. 2, D and E, and fig. S3). We next examined whether vDNA exposure correlates with innate immune activation. Recent studies have shown that capsid stability, as modulated by capsid inhibitors such as LEN, plays a key role in regulating innate immune sensing through the cyclic GMP AMP synthase–Stimulator of Interferon Genes (cGAS-STING) pathway (*50*). In this study, we aimed to investigate whether capsid inhibitors alter HIV-1–MLOs, which are enriched in viral cores within the nucleus, and whether this perturbation promotes cGAS activation through increased exposure of ds vDNA. To address this, capsid inhibitors were added at 48 hours postinfection, a time point at which most of the viral cores are localized within HIV-1–MLOs (*17*). Using real-time PCR to amplify MxA and CXCL10, two interferon-stimulated genes (ISGs) we found that the disassembly of HIV-1–MLOs by high dose of PF74 administered 48 hours postinfection robustly induces MxA (37-fold) and CXCL10 (23-fold) expression compared to untreated controls, consistent with prior reports (*17*). Here, we show that treatment with 50 nM LEN—a concentration observed in plasma during the initial days postinjection—completely disrupts HIV-1–MLOs (fig. S4) and robustly induces ISGs, including a 20-fold increase in MxA and a 16-fold increase in CXCL10. In contrast, 5 nM LEN—a steady-state plasma concentration in treated individuals—induces no detectable ISG response, consistent with the retention of ds vDNA within HIV-1–MLOs (Fig. 2F and fig. S4). The statistical analysis was done using the ordinary one-way analysis of variance (ANOVA) test [MxA ****P < 0.0001, **P = 0.0031, *P = 0.0138, not significant (ns) = 0.6465; CXCL10 *P = 0.0402, *P = 0.0429, ns ≥ 0.9999]. These results support the notion of ongoing vDNA production in viral cores within HIV-1–MLOs, as 50 nM LEN was added after their formation once the virus had already entered the nucleus. The disruption of HIV-1–MLOs by 50 nM LEN induces ISGs expression, similarly to 10 μM PF74.

These results demonstrate that LEN dose of 5 nM preserves HIV-1–MLOs, thereby sequestering vDNA and preventing innate immune activation. In contrast, higher doses of LEN as 50 nM (fig. S4) or PF74 concentrations dismantle HIV-1–MLOs, expose vDNA, and trigger a potent ISG response (Fig. 2F).

### LEN stabilizes nuclear HIV-1 cores and retain vDNA within MLOs

LEN (5 nM) potently inhibits HIV-1 replication without impairing nuclear import or reverse transcription (Fig. 1, A to F), suggesting that loss of infectivity (Fig. 1, G to I) arises from vDNA retention within HIV-1–MLOs containing viral cores (Fig. 2, D and E). To investigate the underlying mechanism, we use cryo–correlative light and electron microscopy (cryo-CLEM) (*51*) and cryo–electron tomography (cryo-ET) on infected THP-1 macrophage-like cells expressing CPSF6-mNeonGreen (Fig. 3A) to target fluorescently labeled CPFS6 puncta and obtain 3D reconstructions of HIV-1–MLOs at molecular resolution. Cells were differentiated on electron microscopy (EM) grids, infected for 72 hours with HIV-1 in the presence or absence of LEN and then vitrified. Cells containing CPSF6 puncta in the nucleus are identified by cryo–fluorescence imaging and further targeted and processed by cryo–focused ion beam milling, followed by cryo-ET imaging of the corresponding nuclear regions (Fig. 3, A and B; movies S1 and S2; and see Materials and Methods for experimental details). The resulting tomograms enabled the visualization of HIV-1–MLO regions containing quasi-intact capsid cores, organized in clusters and randomly oriented, displaying various degrees of electron dense material in their lumen (Fig. 3B and movie S1). Identifying HIV-1 core structures within the nucleus remains challenging, as previous cryo-ET studies reported fewer than 10 cores in intact cells (*16*, *52*). Here, we were able to identify multiple nuclear HIV-1 cores (161) within HIV-1–MLOs, revealing a substantial difference in both their frequency and content between conditions (Fig. 3, C to F). We found 29 cores in untreated cells (18%) across 10 tomograms of the 100 versus 132 cores in LEN-treated cells across 5 tomograms of 100 (82%) (Fig. 3C and movies S1 and S2). In LEN-treated cells, most cores retain viral material, with 80 appear full (60.6%), 30 half-full (22.7%), and 22 empty (16.7%) (Fig. 3D), whereas untreated cells predominantly display empty cores [21 empty (72.4%), 4 half-full (13.8%), and 4 full (13.8%)] (Fig. 3, D and E).

**Fig. 3.**
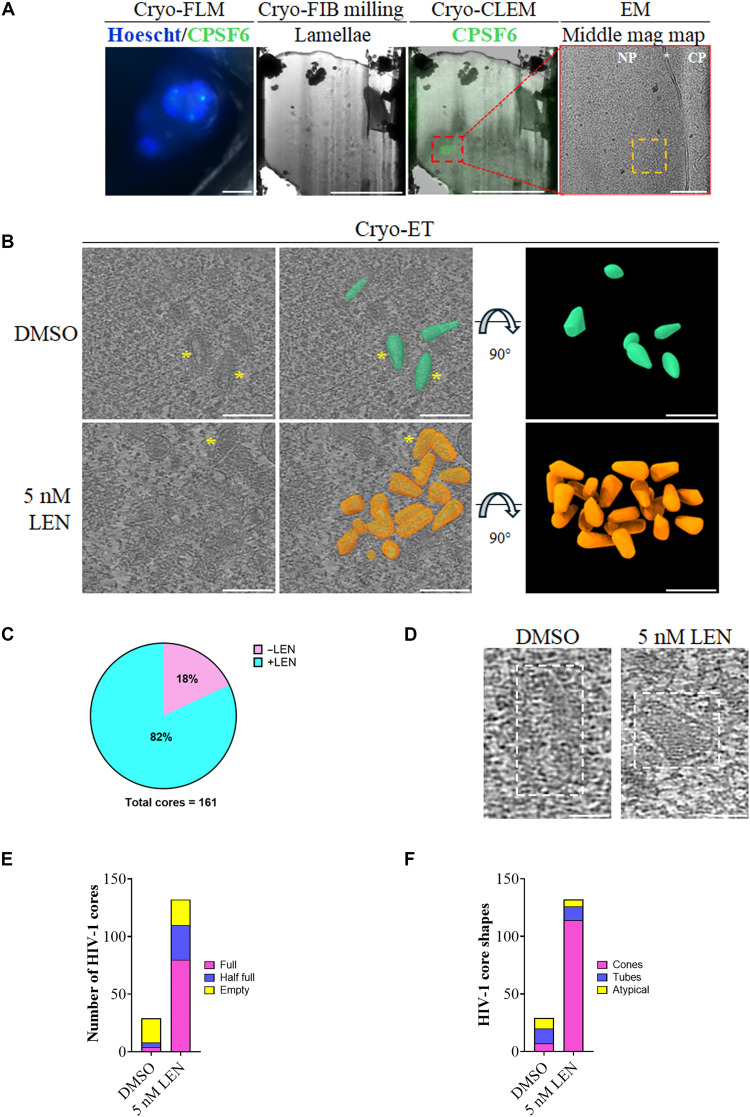
Stabilization of nuclear HIV-1 cores by LEN revealed by cryo-ET. (**A**) THP-1 cells were differentiated on R2/2 Quantifoil EM gold grids and then infected for 72 hours with VSV-G/HIV-1 ∆Env (MOI = 20). After vitrification, a fluorescence map of the entire grid was recorded using both the blue and the green channels for Hoechst 33342 and CPSF6-mNeonGreen, respectively (cryo-FLM). Cells positive for CPSF6 condensates were subsequently milled using cryo–focused ion beam (cryo-FIB). One CPSF6 condensate is highlighted with a red square. The coordinates of CPSF6 condensates identified in the cryo-CLEM step were correlated with SerialEM and tomo5 on the middle magnification EM map (orange square). (**B**) Data were imported to Scipion3 for batch processing. Tomograms containing HIV-1 capsids within the nucleus of differentiated THP-1 cells in presence or absence of LEN (yellow stars). Scale bars, 70 nm. A total of 100 tilt series were recorded for each condition (DMSO, 5 nM LEN). (**C**) Pie chart showing the proportion of cores identified in each condition. A total of 29 cores from untreated cells (pink) and 132 cores from LEN-treated cells (blue) were analyzed. (**D**) Tomograms showing different morphologies of HIV-1 capsids within the nucleoplasm depending on the LEN treatment status. Capsids in both untreated and treated cells are pinpointed with white dashed squares. Scale bars are displayed in white and correspond to 70 nm. (**E**) Histogram displaying the number of capsids full (magenta), half full (purple) or empty (yellow) in the presence or in the absence of LEN. (**F**) Histogram displaying the number of capsids that adopt a conical shape (magenta), a tubular shape (purple), or an atypical shape (yellow) in the presence or in the absence of LEN. Data derive from seven independent experiments. A total of 100 cells per condition were analyzed by cryo-ET.

Core morphology analysis further clarifies the effect of LEN. Whereas in vitro HIV-1 cores are classically conical (*53*–*56*), however, a previous study using a reconstituted system of HIV-1 nuclear import in permeabilized T cells with isolated viral cores observed that tube-shaped capsids more efficiently enter the nucleus (*57*). Here, we directly examined nuclear cores in host nuclei following passage through the nuclear pore complex (NPC) in the presence or absence of LEN. Therefore, the capsids analyzed in our study have undergone the full cytoplasmic trafficking process before nuclear entry, which is not recapitulated in permeabilized systems using isolated cores. In line with the aforementioned study (*57*), in untreated cells, nuclear cores often show noncanonical shapes, with 13 tubular (44.8%) and 9 (31%) atypical (deformed) morphologies predominating and only 7 retaining a conical form (24.1%). In contrast, in LEN-treated cells, most of the cores (114 on 132) preserve their conical architecture (86.4%), with only 12 tubular (9.1%) and 6 atypical (4.5%) (Fig. 3F).

Together, these data indicate that 5 nM LEN stabilizes HIV-1 cores within the nucleus, rather than blocking their entry at the NPC. Maintaining core capsid integrity and delaying the release of viral components shed light on the observed infectivity defect (Fig. 1, G to I). These results indicate that inside HIV-1–MLOs, LEN-induced core stabilization promotes sequestration of vDNA, creating steric hindrance that prolongs the protection of the viral genome in the nucleoplasm from innate immune sensing (Fig. 2, D to F).

### Subtomogram averaging confirms HIV-1 core lattice stabilization in infected cells treated with LEN

The results above suggest that LEN alters or delays the uncoating of the HIV-1 core in the nucleus of infected cells. To test this hypothesis, we perform subtomogram averaging (STA) of hexamers (fig. S5 and movie S3)—the main building blocks of the HIV-1 core lattice ultrastructure (*54*)—to evaluate the state of the observed HIV-1 capsids in the presence and absence of LEN within the nucleus. After a semiautomatic segmentation (*58*–*60*) of cores from tomograms that meet the standard quality for STA (lamellae thickness and vitreous ice), 43 and 23 cores in the presence or in the absence of LEN, respectively, were obtained; 600 points were sampled perpendicular to the surface of each cone (oversampling ∼2.5 times), extracted into subvolumes, and subsequently refined for positions and orientations using wrapper scripts for Tom toolbox (*60*). Duplicates were removed automatically keeping the highest scoring candidates within a set distance threshold. The resulting lattices were manually inspected to confirm the accuracy of recovered positions and orientations and used to reconstruct refined segmentations, where a second round of sampling, alignment, and particle cleaning is performed (movie S3). Remaining particles were then divided into two independent subsets, aligned, and averaged separately to determine the resolution via gold standard Fourier shell correlation (FSC) between half maps (see Materials and Methods and Supplementary Materials for additional details).

In the presence of LEN, the characteristic structure of the HIV-1 hexamer was resolved by STA without applying any symmetry (i.e., C1) at a resolution of 25.7 Å (FSC threshold of 0.143 and 32.6 Å at an FSC threshold of 0.5). The central hexamer together with the six from the surrounding lattice were identified, and an atomic model of the HIV-1 hexamer from the literature was fitted in all hexamers to validate the positioning and size of the retrieved sublattice (Fig. 4A) (*61*, *62*). Similar results are observed when C6 symmetry is imposed during averaging (Fig. 4B), yielding a resolution of 26.7 Å (FSC threshold of 0.5 and 15.6 Å and FSC threshold of 0.143). FSC curves are in fig. S6. In the absence of LEN, the canonical structure of HIV-1 core hexamers is not resolved without imposing C6 symmetry. This is likely because HIV-1 cores were not stabilized by LEN, and the uncoating process generated damaged cores that were insufficient for full structural recovery without treatment. However, imposing C6 symmetry improved refinement to ∼40-Å resolution, allowing partial lattice reconstruction for selected cores (Fig. 4, A and B).

**Fig. 4.**
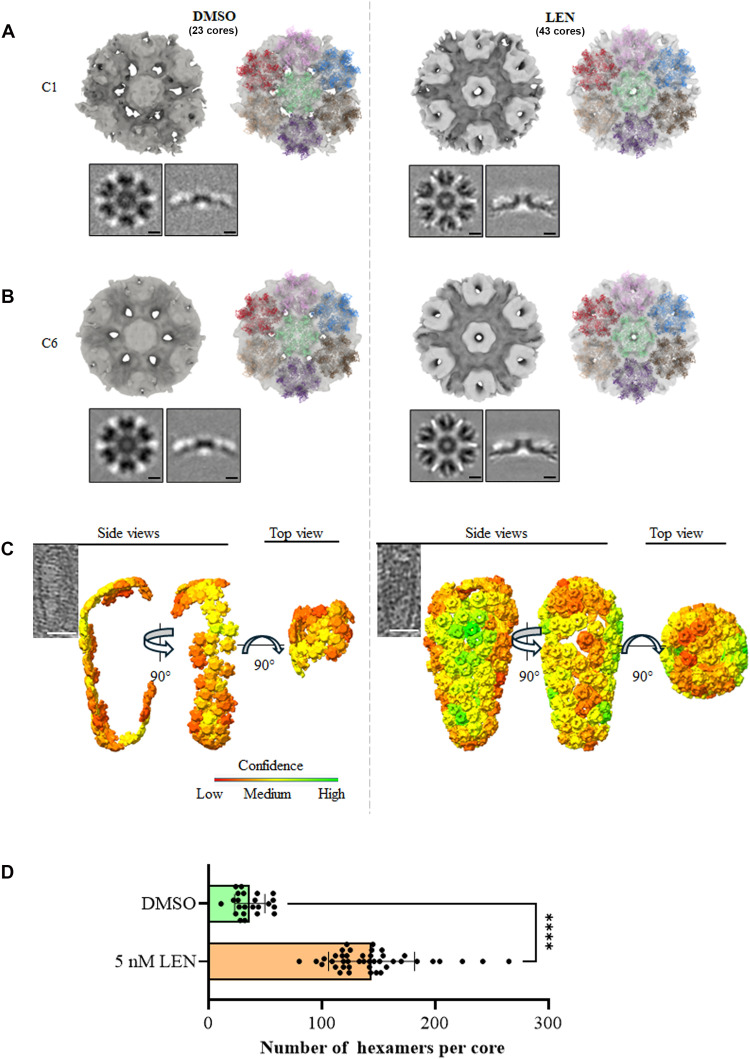
LEN treatment (5 nM) stabilizes and delays HIV-1 nuclear core uncoating in THP-1 cells. (**A**) Structures of HIV-1 nuclear capsid hexamers determined by STA with C1 symmetry in the presence of LEN (right, EMBD-55875) or its absence (DMSO, left). These structures were generated by oversampling and subsequent averaging of 43 cores for the LEN condition and 23 cores for the DMSO control. Fits of seven atomic models of HIV-1 hexamer (Protein Data Bank: 6SKK; shown in green, red, pink, blue, brown, purple, and light brown) into the STA maps are displayed to the right of each model. Scale bars, 50 nm. Reported resolution for the HIV-1 capsid hexamers are 25.7 Å (LEN) and 34.5 Å (DMSO) at FSC cutoff 0.143 between the half maps. (**B**) Structure of HIV-1 nuclear hexamers resolved by STA with C6 symmetry. Resolution: LEN, 25.7 Å (EMDB-55876); DMSO, 37.8 Å (EMDB-55877) at FSC cutoff of 0.5 between the half maps. (**C**) Representative HIV-1 capsids within the nucleus of macrophage-like cells in the presence (right) or absence (left) of LEN. Capsids were reconstructed using two rounds of segmentations and STA (see the Supplementary Materials for details). The color scale indicates the cross-correlations values between the STA average and each retrieved hexamer from the HIV-1 core lattice, ranging from lowest (red) to highest (green). Images are representative of 43 and 23 nuclear cores analyzed in the presence and absence of LEN, respectively. (**D**) Histogram quantifying the number of hexamers recovered within the lattice of HIV-1 cores (43 cores for LEN, orange; 23 cores for DMSO, green). Data are presented as the means ± SD. Statistical significance was assessed using a Welch’s *t* test (****P < 0.0001).

Next, placing the hexamer average into the corresponding tomograms using the positions and orientations obtained through STA revealed that HIV-1 lattice is substantially more complete in the nucleus upon LEN treatment in the infected nuclei at 72 hours postinfection. We observe a mixed population of HIV-1 cores, ranging from nearly complete capsids to those showing some breakage (Fig. 4C and movie S4). On average, 144 recoverable hexamers per HIV-1 core (a single HIV-1 core contains ∼250 hexamers) formed a detectable lattice in the presence of LEN compared to only 36 hexamers per core in the absence of LEN, characterized by extensive uncoating of the HIV-1 cores where only remnant of the viral lattice are detected (Fig. 4D and movie S5). Overall, these results strongly indicate that LEN delays or impairs uncoating, thereby stabilizing the HIV-1 capsid lattice within the nucleoplasm of infected cells.

### LEN alters HIV-1–MLO condensate dynamics and shifts their canonical localization from NSs

CPSF6 binds the viral core shortly after nuclear entry, leading to the formation of HIV-1–MLOs, which typically stabilize within NSs, marked by SC35 (SRSF2), a protein involved in splicing (Fig. 5, A and B) (*40*). Within these NSs, we observe the formation of newly synthesized vDNA (*41*), which is subsequently released for integration into the neighboring chromatin (*39*). To characterize these assemblies, we perform coarse-grained molecular dynamics simulations of biomolecular condensates at amino-acid resolution using the Mpipi-Recharged model (*63*), which represents each residue as a single bead capturing its charge, hydrophobicity, and size while preserving secondary structure and the flexibility typical of intrinsically disordered regions (IDRs). Simulations reveal that CPSF6 together with vDNA forms dynamic MLOs characterized by heterogeneous internal density and high molecular mobility, consistent with a liquid-like condensed phase (Fig. 5C). The introduction of SRRM2 (at an equimolar 1:1:0.1 CPSF6/SRRM2/vDNA ratio)—a key component of NSs (*64*)—stabilized these MLOs by increasing condensate density and strengthening the network of intermolecular contacts within the condensed phase (Fig. 5D). Within these MLOs, all components were homogeneously distributed, consistent with favorable heterotypic interactions that promote colocalization and stabilize the assembly.

**Fig. 5.**
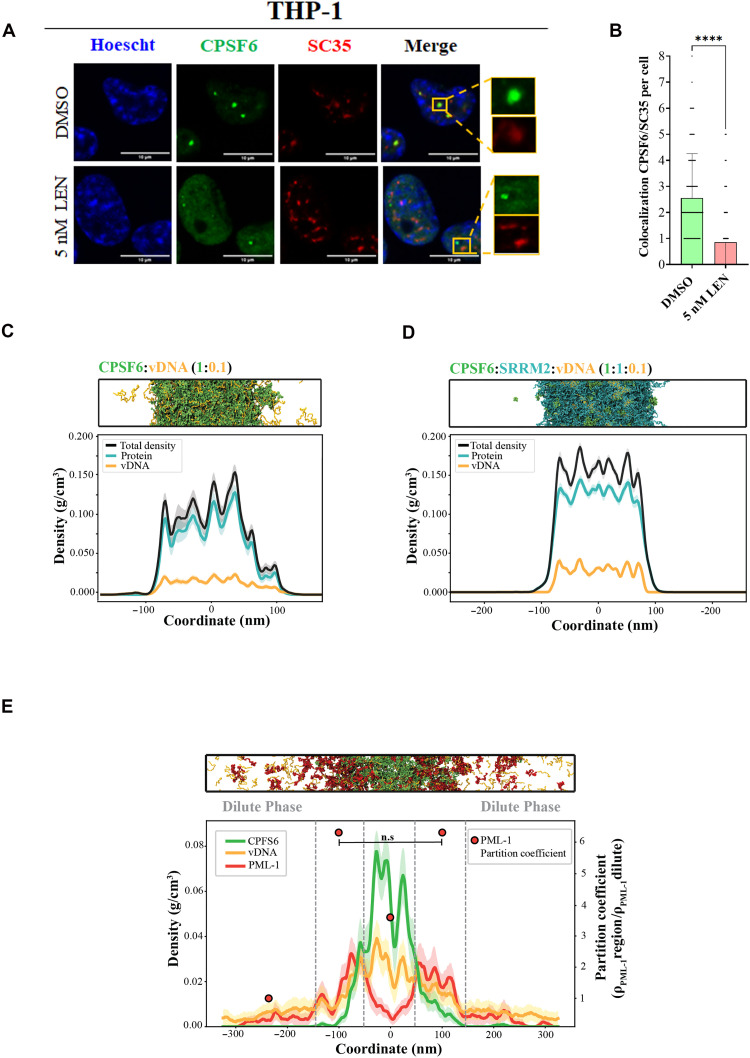
HIV-1–MLOs dissociate from NSs under LEN treatment. (**A**) THP-1 cells were differentiated with PMA and then infected with HIV-1 ∆Env pseudotyped with VSV-G (MOI = 20) for 72 hours in the presence of LEN (5 nM) or left untreated. Cells were then immunostained using antibodies targeting CPSF6 or SC35. Representative immunofluorescence pictures showing the chromatin (Hoechst, blue), CPSF6 (green), and SC35 (red). Scale bars, 10 μm. (**B**) Histogram shows the colocalization between CPSF6 and SC35 upon LEN treatment (red) or without LEN (green). A total of 92 untreated cells and 86 LEN-treated cells were analyzed. Data are presented as the means ± SD and significance was tested using a Welch’s *t* test (****P < 0.0001). (**C**) Direct coexistence simulation of CPSF6 mixed with vDNA at a 1:0.1 amino acid–to–base pair ratio. vDNA chains (orange) partially embed within the CPSF6 phase (teal). (**D**) Three-component simulation of CPSF6 (green), SRRM2 (teal), and vDNA (orange) at 1:1:0.1 stoichiometry. Graphs (C and D) density profiles correspond to total (black), protein (teal), and vDNA (orange) distributions. (**E**) Simulations including CPSF6, vDNA, and PML-1 show that PML-1 (dark red) enriches at the condensate interface rather than the core. CPSF6 is in green and vDNA in orange. Error bars represent standard deviation; *P* < 0.01 except where indicated (ns).

However, in the presence of 5 nM LEN, we observe a significant fraction of HIV-1–MLOs that were spatially independent of NSs, with colocalization dropping from ∼100 to ∼70% (Fig. 5, A and B). To investigate alternative nuclear localizations, we consider studies indicating that transcriptionally competent but silent HIV-1 proviruses localize near PML-NBs, an association linked to repression of viral gene expression (*65*). To test whether PML-1 could interact with HIV-1–MLOs, we performed additional three-component coarse-grained molecular dynamics simulations including CPSF6, vDNA, and PML-1. The analysis of the PML-1 partition coefficient revealed negligible concentration within the interior of the CPSF6-vDNA MLOs, indicating that PML-1 is energetically disfavored within the condensed phase. Instead, PML-1 became enriched at the condensate interface, where it maintained transient interactions with CPSF6. These findings suggest that PML-1 does not cophase separate with CPSF6 and vDNA but preferentially localizes at the CPSF6 MLO periphery, consistent with wetting-like behavior that could facilitate the exchange of vDNA (Fig. 5E). We therefore hypothesized that LEN redirects HIV-1–MLOs from NSs, which are embedded in transcriptionally active SPADs (*66*), toward PML-NBs, which are linked to repressive PML-associated domains (PADs) (*67*). To test this appealing hypothesis in cellulo, we colabel CPSF6, PML, and reverse-transcribed DNA using EdU incorporation, followed by click chemistry. Our data indicate that LEN significantly increase HIV-1–MLOs association with PML-NBs in both THP-1 cells (∼22.5 to ∼52.7%) and primary macrophages (∼10 to ∼27%) (Fig. 6, A to D, figs. S7 and S8). The CPSF6 puncta were often adjacent to, but not colocalized with, PML-NBs, in line with coarse-grained models (Fig. 5E), unlike their tight colocalization with SC35-marked NSs (*40*). Our simulations support the colocalization of CPSF6 condensates with the SC35 marker of NSs (Fig. 5, C and D). SRRM2, which colocalizes with SC35, directly enhances the stability of CPSF6 condensates, allowing them to fuse with NSs (*40*), thereby explaining their tight colocalization in cells, confirmed by modeling (Fig. 5, C and D).

**Fig. 6.**
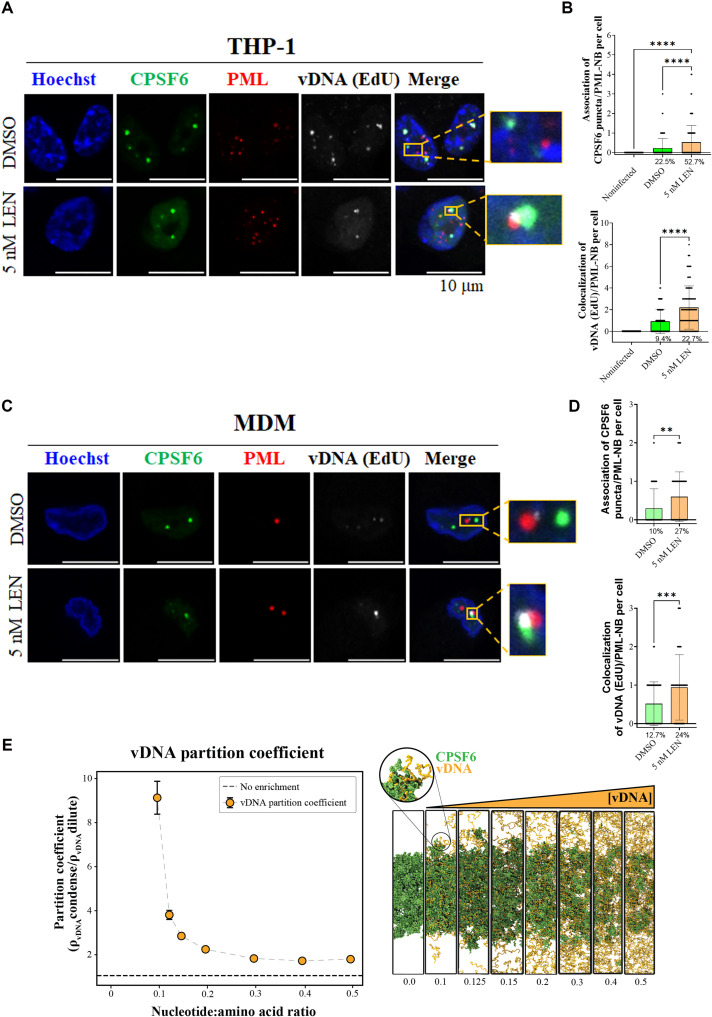
LEN promotes vDNA transfer to PML-NBs. (**A**) THP-1 cells infected for 72 hours with HIV-1 ∆Env VSV-G (MOI = 20) in the presence or in the absence of LEN (5 nM) were processed for both immunostaining and EdU Click-chemistry staining. Z-stacks were acquired with a confocal microscope using the following channels: blue for Hoechst 33342, green for CPSF6, red for PML and far red (white) for the vDNA stained with EdU Click-chemistry. One hundred fifty cells were analyzed in each condition. (**B**) Histograms showing the colocalization between EdU-PML bodies (bottom) and the association of CPSF6-PML bodies (top) per cell. The percentage of cells positive for the respective events (EdU-PML colocalization or CPSF6-PML association) is shown below the histograms. Data are presented as the means ± SD, and statistical significance was assessed using an ordinary one-way ANOVA test (****P < 0.0001). (**C**) Monocytes from healthy donors were differentiated into macrophages and infected with HIV-1 for 72 hours in the presence or in the absence of LEN (5 nM). The panel shows confocal images of fixed primary macrophages immunostained for CPSF6 (green), PML bodies (red), and the vDNA (EdU Click-chemistry, white). (**D**) Histograms showing the quantification of both the colocalization between EdU-PML bodies (bottom) and the association of CPSF6-PML (top) bodies per cell. Data were processed as described in (C) and (D). Welch’s *t* test was used to evaluate the significance of the differences in primary macrophages (***P = 0.0005 and **P = 0.0018). The data are representative of two independent donors. All scale bars are displayed in white and correspond to 10 μm. (**E**) Left: vDNA partition coefficients from direct coexistence simulations. Right: Snapshots showing equilibrium configurations for each titration point, highlighting progressive expulsion of vDNA (orange) from the CPSF6 phase (green) above the saturation threshold.

This is consistent with observations that distinct MLOs rarely fuse due to differences in composition, charge, or material state (liquid-like versus gel-like) (*68*). Instead, they often engage in transient “kiss-and-run” contacts that enable molecular exchange without fusion (*69*). We detect transfer of vDNA from HIV-1–MLOs to PML-NBs, which increases in LEN-treated cells (THP-1: ∼9.4 to ∼22.7%; MDMs: ∼12.7 to ∼24%) (Fig. 6, A to D, and figs. S7 and S8). To understand how this transfer occurs, we performed additional coarse-grained simulations in which the concentration of vDNA was progressively increased while CPSF6 levels were kept constant. Throughout the titration, the overall density of the CPSF6 MLOs remained unchanged. As vDNA concentration increases, its enrichment within the MLOs initially rose sharply and then declines beyond a critical nucleotide-to-amino-acid ratio (Fig. 6E). This transition reflects saturation of the maximal amount of vDNA retained in CPSF6 MLOs, after which excess vDNA is selectively expelled while the MLO itself remains intact. This saturation-dependent expulsion provides a physical mechanism by which CPSF6 condensates can release vDNA without dissolving, offering a plausible explanation for how reverse-transcribed genomes disengage from CPSF6 assemblies as reverse transcription proceeds.

While EdU labels all forms of vDNA generated throughout reverse transcription, including early, intermediate, and late products (*41*), to specifically track completed reverse transcription products, such as the ds vDNA, we use the HIV-1 ANCHOR system (*15*, *39*, *48*) to evaluate whether these ds vDNAs could be transferred from HIV-1–MLOs to PML-NBs. As previously shown, the vDNAs are protected, likely, within stabilized viral cores inside HIV-1–MLOs (Fig. 2, D to F). To visualize them, we added high-dose PF74 at 72 hours postinfection, which allows comparable detection of OR-GFP–labeled dsDNA in LEN-treated and untreated cells (Figs. 2E and 7, A and B). We observe that LEN markedly increases colocalization of ds vDNA with PML-NBs compared to untreated cells. In infected but untreated cells, we detect rare colocalization of only seven ds vDNA signals with PML-NBs across 150 cells, with no colocalization observed in 156 LEN-treated cells. Conversely, following PF74 addition in LEN-treated cells, 45 ds vDNAs colocalize with PML-NBs in 96 cells analyzed (Fig. 7, A and B, and fig. S9). Therefore, viral cores labeled with the fluid-phase marker mCherry-2xCL-YFP-Vpr (*70*) rarely colocalized with PML-NBs, irrespective of LEN treatment, but consistently associated with CPSF6 puncta (Fig. 7, C and D, and fig. S10). In 92 untreated cells and 119 LEN-treated cells analyzed, we observed 172 and 165 viral cores colocalizing with CPSF6, respectively, whereas only 13 and 6 cores colocalize with PML-NBs under the same conditions (Fig. 7, C and D, and fig. S10). This indicates that LEN-stabilized viral cores remain sequestered within CPSF6 puncta, in contrast to vDNA, which is transferred between the two MLOs (Figs. 6, A to E, and 7, A to D). Collectively, our results show that LEN redirects HIV-1–MLOs from NSs to PML-NBs, a compartment linked to transcriptional repression of the virus.

**Fig. 7.**
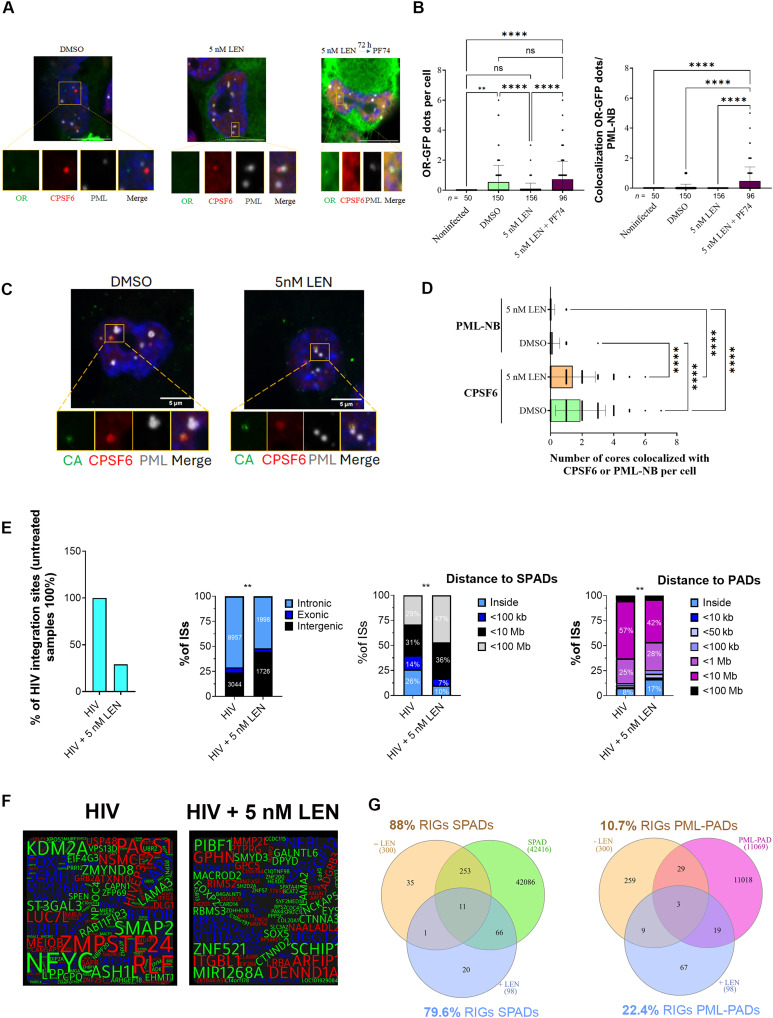
LEN restricts HIV-1 integration and redirects residual viral genomes to PML-PADs. (**A**) THP-1 cells expressing OR-GFP infected with HIV-1 ANCH3 (MOI = 20) and treated, or not, with LEN (5 nM). After 72 hours, CPSF6 puncta were dissolved using PF74 (25 μM). Confocal images show OR-GFP (green, viral dsDNA), CPSF6 (red, HIV-1–MLOs) and PML (white, PML-NBs) after immunostaining using both CPSF6 and PML antibodies. (**B**) Quantification of OR-GFP dots per cell (left) and the number associated with PML bodies (right). Data are presented as the means ± SD and analyzed using an ordinary one-way ANOVA (****P < 0.0001 and **P = 0.0010) (*n* = 50 cells per condition). (**C**) Differentiated THP-1 cells were infected with a YFP-Vpr fluorescent HIV-1 (+/− LEN) and immunostained for CPSF6 and PML: HIV-1 cores (green), CPSF6 (red), and PML (white). (**D**) YFP fluorescent cores that colocalize either with CPSF6 or PML bodies were counted and plotted in a histogram. The means ± SD for each condition is shown (*n* = 100). Statistical test: Ordinary one-way ANOVA test (****P < 0.0001). Scale bars, 10 μm. (**E**) Graph shows the percentage of HIV-1 ISs obtained from libraries generated using genomic DNA extracted from THP-1 cells infected with VSV-G/ΔEnv HIV-1, 3 d.p.i. In untreated samples, a total of 12,424 unique ISs were identified after filtering and reported as 100%. Cells treated with 5 nM LEN yielded 3842 ISs. Percentage of ISs in the overall genomic distribution of ISs with respect to genomic features is shown. (**F**) Word cloud graph showing the top 100 most targeted RIGs in untreated and LEN-treated cells. (**G**) Venn diagram showing the percentage of HIV-1 RIGs associated with SPAD (left) and PML-PADs (right): ISs from THP-1 cells infected with or without 5 nM LEN (blue and orange, respectively) were intersected with published gene sets associated with SPADs (green; left) and PML-PADs (pink; right).

### LEN reshapes HIV-1 IS selection toward silenced chromatin

It has been recently reported that capsid inhibitors, including LEN, can alter HIV-1 integration site (IS) selection, shifting integration away from transcriptionally active regions toward more repressive genomic environments (*71*, *72*). However, whether this redistribution of ISs is linked to changes in nuclear organization and the spatial repositioning of vDNA within macrophages remains unknown. After observing a shift in the nuclear localization of vDNA upon LEN treatment, we decided to investigate whether LEN alters proviral IS distribution. To this end, we performed high-throughput ISs recovery in THP-1 cells infected with a full-length ∆Env HIV-1 virus, in the presence or absence of 5 nM LEN. Genomic DNA was extracted and extensively digested to remove nonintegrated vDNA, particularly abundant in nondividing cells such as macrophages. Viral ISs were recovered by linker-mediated PCR coupled to next-generation sequencing in a protocol extensively used in preclinical studies (*73*–*75*). Sequencing reads were bioinformatically trimmed and mapped into the human genome to retrieve the specific site of viral integration in the human genome, as previously described (*73*). First, we observed a reduction in integration frequency in LEN-treated cells with respect to the control: We retrieved 3842 ISs in LEN-treated cells and 12,641 ISs in control, untreated cells (Fig. 7E and table S1). Then, these integration events follow a noncanonical distribution along the genome. The canonical pattern of HIV-1 integration mostly targeting introns of a particular subsets of cellular genes in a cell-specific fashion, and intergenic regions to a lesser extent (*76*), was correctly reproduced and observed in untreated but infected control cells (Fig. 7E and table S1). In contrast, LEN-treated cells exhibit a marked deviation from the canonical integration pattern, with a substantial shift toward intergenic regions, accounting for up to 45% of integration events—compared to 24% in control cells. This suggests that LEN treatment non only significantly impacts the overall HIV-1 integration efficiency, but also significantly alters the canonical viral preferences of interaction in the human genome. Interestingly, LEN treatment also alters the propensity of HIV-1 to integrate close to SPADs in favor of PML-PADs. In particular, LEN treatment markedly decreases the proportion of ISs within SPADs, from 26% to 10%, and increases their distance from these domains, with 47% of LEN-associated ISs located <100 megabase pairs (Mb) from SPADs compared to only 29% without treatment. Conversely, LEN increases integration events within PML-PADs (17% with LEN versus 8% without). This distribution pattern near PML-PADs is inversely proportional to that observed near SPADs (Fig. 7E).

To gain insight into the differences in integration profiles in cells infected in presence or absence of LEN, we define the sets of genes preferentially targeted by HIV-1 with and without LEN by comparing the actual targeting frequency of each gene with an expected targeting frequency obtained by multiple sampling of a set of random computational ISs, setting the threshold *P* value at <0.01 (with Bonferroni correction for multiple testing). By this analysis, we identify 300 and 98 overtargeted genes, hereon called recurrent integration genes (RIGs), among the 4343 and 2113 genes targeted by the virus in control and LEN-treated cells, respectively. The top 100 overtargeted genes are shown in a cloud graph in Fig. 7F, where the dimension of the gene name is proportional to the targeting frequency. A Gene Ontology (GO) analysis of these overtargeted genes performed with PAN-GO Human Functionome (https://functionome.geneontology.org/) revealed that the ones retrieved in control cells codified proteins involved in histone H3-K36 methylation, peptidyl-lysine demethylation, and the regulation of transcription by RNA polymerase II, while the ones collected in LEN-treated cells were not enriched for any particular functional category (PAN-GO biological process) (table S2).

Cross-referencing HIV RIGs with genes associated with PML-PADs reveal a substantial increase under LEN treatment: 22.4% of RIGs in LEN-treated cells were PML-PAD–associated compared to just 10.7% in untreated conditions (Fig. 7G). Together, these data demonstrate a reduction in viral integration, redirecting the remaining viral genomes toward non-canonical RIGs.

## DISCUSSION

Our findings identify capsid stabilization as a biological mechanism that reprograms the nuclear fate of the HIV-1 genome in macrophages. At 5 nM, LEN preserves core integrity after nuclear entry, redirecting viral genomes away from canonical integration hubs and toward PML-NBs. Previous studies have shown that LEN and related capsid inhibitors can impair HIV-1 nuclear import at subnanomolar to low-nanomolar concentrations in T cell systems, including peripheral blood mononuclear cells (PBMCs) and MT-2 cells (*22*, *29*, *77*). However, differing observations have been reported depending on the experimental approach. For example, biochemical assays have shown that nuclear entry of HIV-1 cores at low-nanomolar concentrations of drug was not altered (*34*), whereas imaging-based studies have reported inhibition of nuclear import under similar conditions (*77*). In addition, a recent work (*71*) demonstrated that IS distribution can be analyzed in the presence of LEN in T cells, supporting the notion that nuclear entry and integration can still occur under these conditions. Together, these findings suggest that the effects of capsid inhibitors on nuclear import are context- and assay-dependent.

Our data indicate that the effect of LEN in macrophages is primarily mediated through the stabilization of the viral capsid, which in turn promotes the persistence of viral complexes within HIV-1–MLOs and modulates vDNA fate. While these results uncover a mechanism by which LEN acts at post-nuclear entry stages in macrophages, additional work will be required to determine how this mechanism operates in other relevant cell types, particularly in CD4^+^ T cells. Notably, previous studies have reported the presence of viral cores within the nucleus of T cells (*16*) and the formation of HIV-1–MLOs (*78*), suggesting that similar effects of LEN on post-nuclear entry processes may also occur in these cells.

We demonstrate that capsid stabilization is sufficient to control viral nuclear trafficking and IS selection, uncovering a spatial layer of viral post-nuclear entry regulation that influences infection outcome. To investigate the structural basis of this phenomenon, we applied cryo-CLEM and STA to reconstruct HIV-1 capsids directly within infected macrophage nuclei. A recent study was able to identify multiple cores in the nucleus of a reconstituted system of HIV-1 nuclear import in permeabilized T cells with isolated viral cores (*57*). However, previous in situ cryo-ET studies captured only a limited number of nuclear HIV-1 cores (*16*, *52*). In contrast, we identified 161 native nuclear cores across just 200 tomograms—a >100-fold increase in detection compared to prior studies in situ studies (*16*, *52*)—enabling statistically robust structural analyses. From this dataset, we obtained high-resolution structural information from 66 nuclear cores under physiologically relevant conditions, allowing direct and reliable comparisons between untreated and LEN-treated infections. In untreated cells, the ultrastructure of nuclear capsids appears more relaxed and frequently lack internal density, indicating partial uncoating. LEN-treated cells, by contrast, display compact capsids with enhanced lattice integrity and retention of electron-dense material—hallmarks of stabilized core structure. These differences support a model in which LEN delays uncoating and maintains viral genome integrity postentry. Despite structural preservation, residual gaps remained in some nuclear cores. Some may reflect absent pentamers that were not captured by our STA pipeline in line with a recent study (*30*), technical limitations related to cryo-ET (such as lack of information in the missing wedge area), or indicate delayed uncoating events. Recent computational studies have shown that LEN binding stabilizes CA hexamers and perturbs the balance between hexameric and pentameric assemblies, thereby influencing capsid curvature and lattice organization (*79*, *80*). However, these conclusions are largely derived from in vitro assembly systems and computational modeling, while the direct visualization of these effects within viral cores in infected cells—particularly following nuclear entry—remains unclear. In this context, our cryo-ET analysis of viral cores within infected macrophages provides complementary in situ observations. Although our approach does not resolve capsid architecture at a level sufficient to capture the pentameric units, the increased number of intact and morphologically preserved cores observed upon LEN treatment is consistent with enhanced capsid stability and altered lattice organization. Alternatively, these holes in viral cores may represent channels for genome release. Supporting this, we observed that following reverse transcription, vDNA accumulates within HIV-1–MLOs until a concentration threshold is reached, at which point excess genomes diffuse outward—a phenomenon predicted by simulations and experimentally confirmed. A previous study has shown that LEN treatment increases HIV-1 integration within lamina-associated domains, which are typically disfavored for viral integration (*29*). Building on this, our study investigates a distinct nuclear subcompartment—specifically, the chromatin environment surrounding PML-NBs. We demonstrate that in LEN-treated cells, vDNA is actively rerouted into PML-NBs, showing a novel spatial reorganization of the HIV preintegration complex within the nucleus. HIV-1–MLOs and PML-NBs remain distinct but closely associated and do not undergo fusion. Instead, molecular exchange occurs between adjacent condensates, consistent with general principles of phase-separated compartmentalization (*81*, *82*). This atypical viral nuclear environment likely delays or limits integration by retaining viral genomes in repressive chromatin microenvironments.

The redirection of viral genomes from active integration zones near NSs to PML-NBs—structures associated with transcriptional repression (*67*, *83*)—without condensate fusion offers an additional plausible mechanism for LEN’s antiviral activity. More broadly, the significantly increased number of analyzable nuclear cores—enabled by LEN’s stabilizing effect—allowed us to detect subtle but consistent spatial and structural differences between conditions. Our work underscores how capsid-targeting antivirals like LEN exploit the physical properties of the viral core to interfere with integration competence. We estimate that prolonged LEN exposure, even at low concentrations, may progressively reduce the pool of integration-competent genomes by redirecting residual replication events away from active chromatin and toward repressive compartments.

On the other hand, while 5 nM LEN effectively modulates IS selection by promoting integration in PADs, such a concentration remains permissive for a low level of HIV integration, potentially allowing escape from complete replication blockade. This raises the question of whether higher therapeutic concentrations might further limit residual integration. However, existing evidence indicates that the continuous presence of LEN at 5 nM during infection and replication kinetics is sufficient to prevent productive infection in long-term cultures of CD4^+^ T cells. Thus, while transient exposure at this concentration might not completely abrogate integration, the persistent presence of LEN appears to maintain control over replication. These observations suggest that the current therapeutic dosing may be justified, not necessarily by maximal suppression of integration events per se but by the sustained inhibition of replication in the presence of the drug. Nonetheless, further pharmacodynamic studies could help determine whether higher LEN concentrations offer additional clinical benefit without compromising safety.

Last, understanding how nuclear compartmentalization and condensate behavior intersect with capsid integrity may open previously unknown avenues for long-acting therapeutic strategies aimed at durable viral suppression or even reservoir depletion.

## MATERIALS AND METHODS

### ISGs quantification by single-step qRT-PCR

The activation of Innate Immunity in presence of Lenacapavir was assessed by measuring the expression of Interferon Stimulated genes (ISGs) by RT-qPCR. THP-1 cells were differentiated in RPMI medium (1,5.10^6^ cells in 2 ml of medium per well of TPP 12-wells plate) supplemented with 10% FBS, Penicillin (100 units/ml), Streptomycin (100 μg/ml) and 160 nM phorbol 12-myristate 13-acetate (PMA). After 48 hours of differentiation, macrophage-like cells were infected with HIV-1 [VSV-G ΔEnv IN(HA), BRU] at MOI = 10 for an additional 48 hours. Subsequently, the medium was removed and replaced by fresh RPMI (10% FBS, antibiotics, and PMA) or RPMI that contains the following drugs: 25 μM of PF74, 50 nM and 5 nM of LEN. The cells were then incubated for an additional 24 hours at 37°C and 5% CO_2_. The RNA molecules were extracted thanks to the RNeasy Mini Kit (QIAGEN, #74104) according to the manufacturer’s protocol. The quantity of resulting RNA was measured with a NanoDrop device (Labtech, #ND-1000). For each condition, 5 ng of RNA was used to perform a single-round reverse transcription quantitative PCR (RT-qPCR) using the SuperScript III Platinium SYBR Green One-Step qRT-PCR kit (Invitrogen, #11736059). The qRT-PCR was performed in 20 μl with a mixture composed of both forward and reverse primers (MxA, CXCL10), 10 μl of SYBR Green, 0.3 μl of SuperScript III/Platinium Taq, 5 μl of RNA, and complete up to 20 μl with diethyl pyrocarbonate (DEPC)–treated water. The qRT-PCR cycle used was as follows: 50°C for 5 min and 95°C for 5 min for cDNA synthesis, 40 cycles (95°C for 15 s, 55°C for 30 s, and 72°C for 30 s) for amplification of target genes and 95°C for 15 s, 55°C for 15 s, 90°C for 15 s for melting curves analysis. All samples were normalized using a pair of oligonucleotides targeting the ACTIN gene. Results are displayed as fold increase compared to the noninfected condition. All oligonucleotides are listed in table S3.

### Viral production and titration

HEK293T human embryonic kidney cells (American Type Culture Collection) were used to produce viruses. The nonreplicative VSV-G ΔEnv HIV-1 (BRU) IN(HA) virus where the integrase (IN) protein is fused to the HA tag was produced by cotransfecting the human embryonic kidney (HEK)–293T cells through calcium chloride coprecipitation with the HIV-1 (BRU) ΔEnv IN(HA) plasmid with the pCMV–VSV-G plasmid and pSIV3^+^ plasmid encoding for the SIVMAC protein Vpx. Replicative HIV-1 viruses were produced by transfection of the cells with an HIV-1 plasmid AD8. CMV-OR-GFP lentiviral vector (LV) was produced using a second-generation packaging system: 6 × 10^6^ cells were cotransfected in dishes of 10 cm in diameter with the transfer vector (10 μg per cell), the pSD-GP-NDK packaging plasmid encoding for codon-optimized gag-pol-tat-rev (10 μg per cell), and the pCMV–VSV-G envelope plasmid (2.5 μg per cell). HIV-1 Δdelta Env ANCH3 tagged virus pseudotyped with VSV-G as previously described (*15*). The VSV-G ΔEnv HIV-1 pNL43 VPR–yellow fluorescent protein (YFP) (*70*) virus where the Viral Protein R (Vpr) protein is fused to the YFP tag (Addgene, #105215) was produced by cotransfecting the HEK293T cells with the HIV-1 G ΔEnv HIV-1 pNL43 (7.5 μg) plasmid, with mCherry-2xCL-YFP-Vpr plasmid (2.5 μg), with the pCMV-VSV-G plasmid (2.5 μg), and with VPX plasmid (3 μg). Then, the supernatant was collected 48 hours post-transfection of the HEK293T cells, and lentiviral particles were concentrated by ultracentrifugation (22,000 rpm) at 4°C for 1 hour before being stored at −80°C. All viruses and lentiviral vectors were tittered by qPCR using primers specific to the U5/R (vDNA) and the CD3 (cellular DNA) sequences (*17*). LVs and HIV-1 viruses were tittered in 293T cells or THP-1 cells. The viral input, expressed in transducing units (TU) determined by titration, was calculated as the product of the cell number and the desired MOI. All work with nonreplicative HIV-1 virus was performed under Biosafety Level 2 Plus (BSL2+) laboratory conditions.

### p24 dosage by ELISA

To assess the impact of Lenacapavir on the infectivity of a replicative HIV-1 virus we performed a p24 dosage by enzyme-linked immunosorbent assay (ELISA) at 3 days postinfection. To do so, THP-1 cells were allowed to differentiate 48 hours in the presence of PMA (160 nM). Once differentiated, cells were infected with HIV-1 (NL4.3-AD8) at MOI = 5 in the presence or in the absence of Lenacapavir (0.5, 5, and 50 nM). As a negative control, THP-1 cells were left untreated. After 24 hours, the medium was changed. Untreated cells were left without any drugs, and Lenacapavir was maintained for an additional 48 hours. The supernatant was recovered and analyzed with the Lenti-X p24 Rapid Titer Kit (Takara, #632200).

The fitness of HIV-1 (NL4.3-AD8) was also assessed for 21 days in the presence or in the absence of Lenacapavir. First, PBMCs were isolated through Ficoll gradient from healthy donors’ blood, provided by ICAReB-biobank. Primary CD4^+^ T cells were selected with human CD4 Microbeads (Miltenyi Biotec, #130-045-101) from the resulting PBMCs and then resuspended in RPMI supplemented with 10% fetal bovine serum (FBS), 1% antibiotics, and interleukin-2 (IL-2). Twenty-four hours later, CD4^+^ T cells were activated using the Human T Cell Activation/Expansion Kit (Miltenyi Biotec, #130-091-441) and human IL-2 (100 IU/ml; Peprotech, #17881063) at a density of 2.10^6^ cells/ml in the same supplemented RPMI medium for 72 hours. Following the activation, CD4^+^ T cells (density of 2 × 10^6^ cells/ml) were plated in TPP 96-well plate and infected with HIV-1 (pNL4-3-AD8) at MOI = 0.5 in the presence of Lenacapavir in supplemented RPMI plus IL-2. Infected CD4^+^ T cells without Lenacapavir were used as positive control. At days 0, 3, 7, 10, 14, and 21, the supernatants were collected and subjected to Lenti-X p24 Rapid Titer Kit ELISA. To ensure the survival of CD4^+^ T cells over time, the medium was changed every 3 days for the same supplemented RPMI medium.

The p24 ELISA using the Lenti-X p24 Rapid Titer Kit was performed according to the manufacturer’s instructions. Briefly, HIV-1 capsids were allowed to bind on p24 antibody precoated eight-well strips for 1 hour at 37°C. Then, a biotin-conjugated p24 antibody was added and incubated 1 hour at 37°C. Streptavidin–horseradish peroxidase was added in each well for 30 min at room temperature. Between steps, eight-well strips were washed six times with the wash buffer. Last, 3,3′,5,5′-Tetramethylbenzidine (TMB) substrate solution was added for 30 min at room temperature, and the plate was protected from light with an aluminum foil. The reaction was finally stopped with the stop solution, and the presence of p24 in each well was quantified by measuring the absorbance at 450 nm using a plate reader.

### Immunofluorescence microscopy

THP-1 cells were seeded on 12-mm coverslips (5 × 10^5^ cells/ml) and differentiated with 160 nM PMA for 48 hours. Then, cells were infected with HIV-1 [VSV-G ΔEnv IN(HA), BRU] at MOI = 20 for 72 hours and left either untreated or treated with the following drugs: LEN (0.5 nM, 5 nM, 50 nM, or 5 μM), NEV (10 μM), PF74 (1.25 μM), or RAL (20 μM). Following the infections, cells were washed twice with phosphate-buffered saline (PBS) and fixed with 4% paraformaldehyde for 15 min. After two PBS washes, the remnant of free formaldehyde molecules was quenched with 0.15% glycine for 10 min. Subsequently, cells were permeabilized with 0.5% Triton X-100 for 25 min at room temperature prior to be blocked with 1% PBS–bovine serum albumin (BSA) (30 min). Antibodies incubations were performed at room temperature in a dark humid chamber for 1 hour and 45 min with primary and secondary antibodies, respectively. Washes and antibody dilutions were done in a 1% PBS BSA solution. For primary antibodies, the dilution was prepared as follow: CPSF6 (1:400; Novus Biologicals, #NBP1-85676), HA (1:500; Roche, #11867423001), and PML (1:50; Santa Cruz Biotechnology, #sc377390). For secondary antibodies, the dilution was prepared as follow: donkey rabbit Cy3 (1:1000; the Jackson Laboratory, #711-165-152), goat rabbit Alexa Fluor 488 (1:300; Invitrogen, #A32731), and goat mouse Alexa-647 (1:300; Invitrogen, #A21235). Images were acquired with an upright confocal microscope (LSM700, Zeiss) with a 63× Plan Apochromat oil immersion objective [numerical aperture (NA) = 1.4].

### Real-time PCR

LRTs, 2LTRs production and HIV-1 vDNA integration in differentiated THP-1 were analyzed by quantitative PCR. Three days after HIV-1 [VSV-G ∆Env IN(HA), BRU] infection (MOI = 20), THP-1 genomic DNA was extracted in each condition using QIAGEN’s QIAamp DNA Micro Kit. The DNA extraction was performed according to the section “Isolation of Genomic DNA from small volumes of blood” from the manufacturer’s protocol. Subsequently, the quantity of DNA was measured under ultraviolet light using a NanoDrop Spectrophotometer (Labtech, #ND-1000). For LRT qPCR, the genomic DNA was treated for 1 hour at 37°C with 20 U of Dpn I (Thermo Fisher Scientific, #ER1705). LRT products were amplified using 60 ng of genomic DNA, the LRT probe (0.1 μM) plus MH531 (0.3 μM), and MH532 (0.3 μM) primers. The amplification cycle was setup as follows: 2 min at 50°C, 15 min at 95°C, and 40 cycles (15 s at 94°C, 30 s at 58°C, and 30 s at 72°C). The standard curve was generated with serial dilutions of the plasmid coding for HIV-1 genome (from 5 × 10^6^ molecules/μl to 64 molecules/μl) to quantify the number LRT in each condition. The quantification of 2-LTRs was performed as described above with the following modifications: The qPCR was done using the 2-LTR probe plus MH535 and MH536 primers; the standard curve was made using the pUC2LTR plasmid (from 10^7^ molecules/ml to 128 molecules/μl). The Integration of HIV-1 vDNA in THP-1 was monitored by Alu-PCR (*9*, *10*). Briefly, integrated vDNA was amplified using primers that bind to the Alu (0.5 μM) sequences from the host genome and the U5 region (LAMBDA U5 at 0.5 μM) of the full vDNA. Proviruses were amplified in each condition with the Platinum SuperFi DNA polymerase (Invitrogen, #12351010) using the following PCR cycle: 2 min at 95°C, 18 cycles (10 s at 95°C, 10 s at 60°C, and 5 min at 68°C), and 5 min at 68°C. Several dilutions of DNA coming from both infected and uninfected cells were amplified with Alu and U3 primers and subsequently used as standard curve. The resulting amplicons were diluted 10 times and quantified by TaqMan qPCR. Integrated vDNA was amplified with a specific probe that recognizes the proviral LTR (0.1 μM) and primers against the LTR (0.3 μM) using the same qPCR cycle described above. Quantitative PCRs were normalized by the amplification of the Actin gene. All oligos used in this study are listed in table S3.

### Flow cytometry

3 days after infection with (pNL4-3 ∆env ∆Nef IRES GFP), cells were treated with 0.1% PBS-EDTA. Then, cells were detached from the plastic substrate by applying a trypsin treatment of 30 min. Once detached, cells were fixed with the BD CellFix solution (Thermo Fisher Scientific, #12756879) for 10 min. Last, cells were analyzed with an Attune NxT Flow Cytometer to evaluate the level of GFP expression. FlowJo was used to process the data.

### EdU/Click-chemistry to stain vDNA

For the staining of vDNA with the EdU/Click-chemistry technology, THP-1 cells were differentiated (48 hours) on 12-mm coverslips using 160 nM PMA. Then, macrophage-like cells were infected with HIV-1 [VSV-G ∆Env IN(HA), BRU] at MOI = 20 for 72 hours in presence of EdU (5 μM) which is a thymine structural analog. After infection, cells were washed once with PBS and subsequently fixed with 4% paraformaldehyde. Fixed cells were washed twice with PBS prior to be washed with a mixture of 3% PBS-BSA. After a permeabilization with a solution of 0.5% Triton X-100 (in PBS) for 20 min, cells were washed once again with 3% PBS-BSA. Coverslips were moved in a dark humid chamber and incubated for 30 min with the Click-iT Plus reaction cocktail (Thermo Fisher Scientific, #C10640) according to the manufacturer’s protocol. Following the Click-chemistry, cells were washed five times with 1% PBS-BSA prior to be blocked with 3% PBS-BSA for 15 min. After five washes to remove the blocking buffer, cells were incubated with CPSF6 primary antibody (dilution 1:400) for 1 hour in 1% PBS-BSA. Five washes with 1% PBS-BSA were made to remove unbound primary antibody, and coverslips were subsequently incubated with the secondary antibody (Alexa Fluor 488 anti-rabbit, dilution 1:1000) in 1% PBS-BSA for 45 min. The incubation of both primary and secondary antibodies were performed at room temperature in a dark humid chamber. After the immunostaining, coverslips were washed five times with PBS, and the chromatin was labeled with Hoescht 33342 (dilution 1:10,000 in water) for 5 min. Last, all coverslips were mounted on microscopy slides using the ProLong Diamond antifade mounting medium. Data were collected using a Zeiss LSM700 upright confocal microscope supplied with a 63× Plan Apochromat oil immersion objective (NA = 1.4). Fluorophores were excited using the following lasers: 405 nm (Hoescht, chromatin), 488 nm (Alexa Fluor 488, CPSF6), and 639 nm (Click-chemistry, vDNA).

### Labeling of full HIV-1 vDNA

The fate of complete ds vDNA was assessed using the HIV-1-ANCH technology (*15*). THP-1 cells expressing the protein OR-GFP were counted using the LUNA system as described above. THP-1 cells were diluted in RPMI medium to reach a density of 5 × 10^5^ cells/ml and then differentiated in 35-mm ibidi m-Dish by adding 160 nM PMA. After 48 hours of differentiation, macrophage-like cells were infected for 3 days with HIV-1∆envIN_HA_∆Nef ANCH3 (MOI = 20) carrying the ANCH3 sequence at the 3′ end of the virus genome. During the infection, cells were either left untreated or treated with a panel of drugs affecting different steps of the replication cycle (0.5 and 5 nM LEN, 10 μM NEV, and 20 μM RAL). Seventy-two hours postinfection, cells were washed once with PBS and incubated in RPMI supplemented with 10% FBS, antibiotics, and Hoescht 33342 (diluted up to 1:40,000). Then, ibidi dishes were transferred inside the chamber of the Ti2E inverted microscope (Nikon) that was previously warmed up at 37°C. Data were recorded using a spinning disk (CSU-W1, Yokogawa) and a 60× objective (Plan Apochromat, oil immersion, NA = 1.4). For CPSF6 puncta blow out, THP-1cells were prepared as described above except that after 72 hours postinfection, cells were washed once with PBS and incubated with RPMI medium that contains 25 μM PF74 for 8 hours. Last, cells were imaged as described above in immunolabeling section. For immunostaining after CPSF6 blow out, cells were processed as described above with the following antibodies: CPSF6 (1:400; Novus Biologicals, #NBP1-85676), PML (1:50; Santa Cruz Biotechnology, #sc377390), rabbit Cy3 (1:1000; the Jackson Laboratory, #711-165-152), and goat mouse Alexa Fluor 647 (1:300; Invitrogen #A21235)

### THP1 vitrification and cryo-FLM

Two hundred mesh electron microscopy gold grids (quantifoil) coated with R 2/2 carbon films have been positioned in 60-mm petri dishes. THP-1 cells (4 × 10^6^) expressing CPSF6 mNeonGreen were seeded and subsequently differentiated using 160 nM PMA. After 48 hours of differentiation, cells were infected with HIV-1 [VSV-G ∆Env IN(HA), BRU] at MOI = 20 for 72 hours, and treated or not, with 5 nM Lenacapavir. Subsequently, fresh RPMI containing 10% FBS, antibiotics, and Hoescht 33342 (dilution 1:40,000) was added to the cells and incubated for 1 hour at 37°C. Infected cells were chemically fixed for 2 hours in PHEM buffer [60 mM Pipes, 50 mM Hepes, 10 mM EGTA, and 2 mM MgCl_2_ (pH = 7.2)] containing 4% formaldehyde and 0.2% glutaraldehyde. Infected macrophage-like cells were vitrified by plunge freezing using an EM GP device (Leica microsystems). The parameters used for vitrification were as follows: liquid ethane at −180°C, temperature at 20°C, and humidity at 90%. A total of 4 ml of PBS was added on each grid before 6 s of blotting from the backside of the grid using a Whatman filter paper. Following the vitrification, grids were clipped in autogrid rings using a clipping station (Thermo Fisher Scientific). The grids were then inserted in a cryo-CLEM microscope (Leica Microsystems) equipped with a 50× objective for Cryo-FLM. The temperature of the stage was maintained at −180°C using liquid nitrogen. Z-stacks (25 μm) of the milling area were recorded with the LAS X software in the following channels: blue for Hoescht 33342, green for CPSF6 mNeonGreen, and the reflection. Cryo-CLEM data were processed with the LasX software, where maximum projection intensity was applied on relevant Z-stacks.

### Cryo-FIB milling and cryo-ET data acquisition

Vitrified THP-1 cells were cryo-FIB milled in an Aquilos (Thermo Fisher Scientific) dual beam cryo-FIB microscope. The atlas of each AutoGrid was recorded with the Maps software (Thermo Fisher Scientific) at low magnification (×155) using the electron beam set at 2 kV with a current at 25 pA. The resulting Atlases were aligned with the corresponding fluorescence maps using the Maps software using a three-point alignment procedure. Then, on each infected cells, a lamellae site was manually added. The gas injection system was used to coat for 90 s the AutoGrids with a layer of organometallic platinum. The preparation (eucentricity, electron and ion beams coincidence, milling angle of 10°, and positioning of the milling box) was done manually at each lamellae site. The milling procedure was performed with the AutoTEM software (Thermo Fisher Scientific) using a 30-kV ion Gallium beam. Lamella milling was done in a stepwise manner by reducing the thickness of the sample as follows: After engraving of stress relief cuts, a 5 μM lamella was generated with a 0.3 nA current (rough milling). The lamella thickness was reduced to 2.5 μM with the same current (medium milling). The fine milling was performed at 0.1 nA to reach a lamella thickness of 1 μM. A finer milling step was applied to reduce the lamella thickness to 500 nm with a current of 50 pA. Last, the polishing was performed manually at 30 pA to reach an expected size of 200 nm.

After cryo-FIB milling the AutoGrids were inserted in a cryo-confocal STELLARIS 8 microscope (Leica microsystems) cooled down at −180°C to perform the step of CryoCLEM on lamellae. Mini Z-stacks (5 μM) of each Lamella were recorded on both the blue channel (Hoescht, 33342) and the green channel (CPSF6-mNeonGreen). Z-stacks were recorded with the following parameters: pinhole at 1, zoom factor of 2, pixel size of 108 nm, and dwelling time of 875 ns. Each Z-stacks were processed with maximum projection intensity using the LAS X process tab.

Cryogenic electron tomography data collections were performed with a 300 kV Titan Krios G3 microscope equipped with a cold field emission gun (FEG), a Falcon 4i direct electron detector (counting mode), and a Selectris X imaging filter. All maps and tilt series were acquired with the SerialEM software (version 4.2). Atlases of whole grids were recorded at low magnification (×155). Then, individual montages [middle magnification maps (MMMs)] of each lamella were recorded in low dose mode at a magnification of ×8 to ×700 with an exposure time of 1 s and a spot size of 5. The fluorescence map of each lamella (one step above) was superimposed on the corresponding MMM using eight registration points and the option transform items of SerialEM. Then, on each CPSF6-mNeonGreen fluorescent point, tilt series were acquired with SerialEM and Tomo5 (Thermo Fisher Scientific software) in low-dose mode. Projection images were recorded at a magnification of ×53,000 that corresponds to a nominal pixel size of 2.445 Å. Tilt series were acquired following a dose symmetric scheme starting from −50° to +70° with an increment of 3° that results in 41 projection images per tilt series. The total dose was set up at 180 e^−^/Å^2^ (dose rate of 4 to 6 e^−^/pixel/s on the lamellae) with a variable exposure time to be 1.6 time higher at 60°. The nominal defocus during tilt series acquisition was set to vary between −2.0 and −4.0 μm. The energy slit width used was 20 eV.

### CD14^+^ cells isolation from the blood of healthy donors and macrophage differentiation

Human peripheral blood samples were collected from healthy volunteers through the Clinical Investigation Center INVOLvE (Investigation and volunteers for human health) of the Institute Pasteur. The participants received oral and written information about the research, and gave written informed consent in the frame of the COSIPOP cohort after approval of the CPP Est II Ethics Committee (20 February 2023). The blood of each donor was diluted in a solution of 2% PBS-FBS (vol/vol) and SepMate tubes (STEMCELL Technologies) were filled with 15 ml of Ficoll. The mixture of blood 2% PBS-FBS was poured in a SepMate tubes and then centrifuged at 1200*g* for 10 min. PBMC cells were recovered (white ring after the spin) and diluted in 50 ml of 2% PBS-FBS. PBMC cells were centrifuged at 300*g* for 8 min before be resuspended in 50 ml of 2% PBS-FBS. Cells were again centrifuged for a second round at 300*g* for 8 min, and the resulting pellet was resuspended in 50 ml of magnetic-activated cell sorting (MACS) buffer. To continue cells were counted thanks to the LUNA system by mixing 1 ml of orange acridine and 9 ml of PBMC cells. The remaining cells were centrifuged at 300*g* for 6 min, and the pellet was resuspended in MACS buffer (80 ml for every 10^7^ cells). a-CD14 MACS MicroBeads were added to the cell mixture (20 ml for every 10^7^ cells). The solution of anti-CD14 MicroBeads and cells were incubated at 4°C for 15 min. After being centrifuged at 300*g* for 6 min, PBMC cells were resuspended in 500 ml of MACS buffer. CD14^+^ cells were finally sorted using MACS columns for magnetic cells separation according to the manufacturer protocol. Cells were counted once again as described above. Before their differentiation, sorted cells were washed twice with RPMI medium supplemented with 10% FBS. CD14^+^ monocytes were resuspended in RPMI containing 10% FBS and macrophage colony-stimulating factor (20 ng/ml) (Myltenyi, 130-096-491) to reach a density of 500,000 cells/ml. A 1 ml of cell mixture was added in TPP 24-well plates where coverslips were previously dropped in the bottom of each well. After 3 days of differentiation, the medium was removed and fresh RPMI (10% FBS plus antibiotics) was added on cells for 24 hours. Then, cells were infected with HIV-1 [VSV-G ∆Env IN(HA), BRU] at MOI = 20 for 72 hours in the presence or in the absence of 5 nM LEN. Cells were chemically fixed and processed for Click-chemistry and immunolabeling as described above. The following antibodies were used: CPSF6 1:400 (Novus Biologicals, #NBP1-85676), PML 1:50 (Santa Cruz Biotechnology, #sc377390), goat anti-rabbit Alexa Fluor 488 (1:300; Invitrogen, #A32731), and mouse cy3 (1:1000; Jackson ImmunoResearch, #715-165-150).

### Model and force field

All simulations were performed using the Mpipi Recharged coarse-grained model (*63*), which represents proteins and nucleic acids at a residue-level resolution, retaining their chemical specificity through residue-specific hydrophobic and electrostatic interactions. Each amino acid is represented as a single bead with a specific charge, excluded volume radius, hydrophobicity, and mass (*84*). The beads are positioned in the Cα of the amino acid backbone. This model comprises 40–amino acid types: 20 for the structured part of the protein and 20 for IDRs. The structured part’s amino acids are enforced to maintain their secondary structure via rigid-body interactions. The force field is defined as

$$E=E_{\text{Bonds}}+E_{\text{Electrostatics}}+E_{\text{Hydrophobic}}$$

The bonded potential follows a harmonic potential, given by

$$E_{\text{bonds}}=\sum_{i}\frac{1}{2}k{\left(r_{i}-r_{i,\text{ref}}\right)}^{2}$$

where, $k=9.6\mathrm{kJ}\cdot {\mathrm{mol}}^{-1}\cdot {Å}^{-2}$, $r_{i}$ is the distance between bonded particles and $r_{i,\text{ref}}=3.81Å$ is the equilibrium bond length between Cα. Electrostatic interactions between amino acids is modeled using a Yukawa potential

$$E_{\text{Electrostatic}}=\sum_{i}\sum_{j<i}\frac{A_{\mathrm{ij}}}{r}e^{-r\kappa }$$

where $A_{i,j}$ defines the interaction strength between specific charged amino acid pairs, r the distance between interacting residues, and the Debye length κ modulates the ionic strength. The sum is over all nonbonded bead pairs. The relation between salt monovalent concentration and κ is defined by $\kappa =\sqrt{8\pi BC_{s}}$, where *B* is the Bjerrum length and $C_{s}$ is the salt concentration.

Last, short-range interactions were parameterized using the Wang-Frenkel pair potential, which includes excluded volume repulsion and short-range attraction. This potential, ϕ, between beads i j separated by a distance r is described by

$$E_{\text{Hydrophobic}}=\Phi \left(r\right)=\varepsilon_{\mathrm{ij}}\alpha_{\mathrm{ij}}\left[{\left(\frac{\sigma_{\mathrm{ij}}}{r}\right)}^{2\mu_{\mathrm{ij}}}-1\right]{\left[{\left(\frac{R_{\mathrm{ij}}}{r}\right)}^{2\mu_{\mathrm{ij}}}-1\right]}^{2\upsilon_{\mathrm{ij}}}$$

where

$$\alpha_{i,j}=2\nu_{i,j}{\left(\frac{R_{\mathrm{ij}}}{r}\right)}^{2\mu_{\mathrm{ij}}}{\left\{\frac{1+2\upsilon_{ij}}{2\upsilon_{ij}\left[{\left(\frac{\sigma_{ij}}{r}\right)}^{2\mu }-1\right]}\right\}}^{2\upsilon_{\mathrm{ij}}+1}$$

Here, r is the distance between i,j particles and σ is the excluded volume. The cutoff of the potential between that particular amino acid pair is $R_{i,j}=3\sigma_{\mathrm{ij}}$. The exponents $\mu_{i,j}$ and $\nu_{i,j}$ are also pair-specific and the values of $\sigma_{i,j}$ and $\varepsilon_{i,j}$. The buried interactions of the globular domains’ residues are scaled by 0.7 a factor and $\sqrt{0.7}$ between a globular amino acid and an IDR residue. We use a $3\sigma_{\mathrm{ij}}$ cutoff for short-range interactions and 3.5 nm for long-range interactions.

Software and hardware: Simulations used the Mpipi implementation in OpenMM 8.1.2 with GPU acceleration on NVIDIA H100 GPUs (CUDA, double precision) and 20 CPU cores at Marenostrum 5 at the Barcelona Supercomputing Center (BSC). Analyses were performed in Python 3.9 using OpenMM-tools, NumPy, Pandas, SciPy, Matplotlib, MDTraj, tqdm, and Seaborn. Visualization used VMD 1.9.4a55 (*85*) with Tachyon rendering on NVIDIA RTX 4070 GPU.

### IS distribution and bioinformatic analysis

Briefly, THP-1 cells were infected and maintained in culture for 3 days. The genomic DNA was extracted, and 1 μg was digested overnight at 37°C with Dpn I [DNA (20 U/μg)] to remove residual plasmid DNA from viral preparation. LTR viral-genome junctions were amplified by ligation-mediated PCR, sequenced, and bioinformatically trimmed as described in (*73*). ISs were mapped onto the hg19 genome assembly. Comparative GO analysis was performed with PAN-GO enrichment tool available at https://functionome.geneontology.org/. Functional categories were considered significantly enriched if having a false discovery rate < 0.05 and a *P* value < 0.001. To perform analysis of ISs distances from SPADs and PML-PADs (*67*, *86*), the publicly available datasets of SPAD and PAD chromosomal regions (Gene Expression Omnibus Database at GSE248644 (GSE248644_SRR3538917_95P.txt.gz) and GSE226210 (GSM7062241, GSM7062242, and GSM7062243) have been used. The Venn diagram has been generated as follows: The SPAD genes used for the analysis were obtained from table S3 in (*66*). PAD genes were derived from table S3 (“Genes falling in PADs”) in (*67*). These gene sets were intersected with the 300 RIGs identified in the absence of LEN and the 98 RIGs identified in the presence of LEN.

## Supplementary Materials

### The PDF file includes:

Figs. S1 to S10 Tables S1 to S3 Supplementary Methods Legends for figs. S1 to S10 Legends for movies S1 to S5
