# Supplementary material for: Capsid stabilization reprograms the nuclear fate of the HIV genome
Source: Sci Adv. 2026 Aug 7;12(32):eaef9469. doi: 10.1126/sciadv.aef9469 (PMC13450216; doi:10.1126/sciadv.aef9469)
Supplement: Supplementary file 1 [file sciadv.aef9469_sm.pdf]

Science Advances
AAAS

Supplementary Materials for

Capsid stabilization reprograms the nuclear fate of the HIV genome

Thierry Mourer *et al*.

Corresponding author: Francesca Di Nunzio, dinunzio@pasteur.fr

Sci. Adv. **12**, eaef9469 (2026)
DOI: 10.1126/sciadv.aef9469

The PDF file includes:

Figs. S1 to S10
Tables S1 to S3
Supplementary Methods
Legends for figs. S1 to S10
Legends for movies S1 to S5

Other Supplementary Material for this manuscript includes the following:

Movies S1 to S5

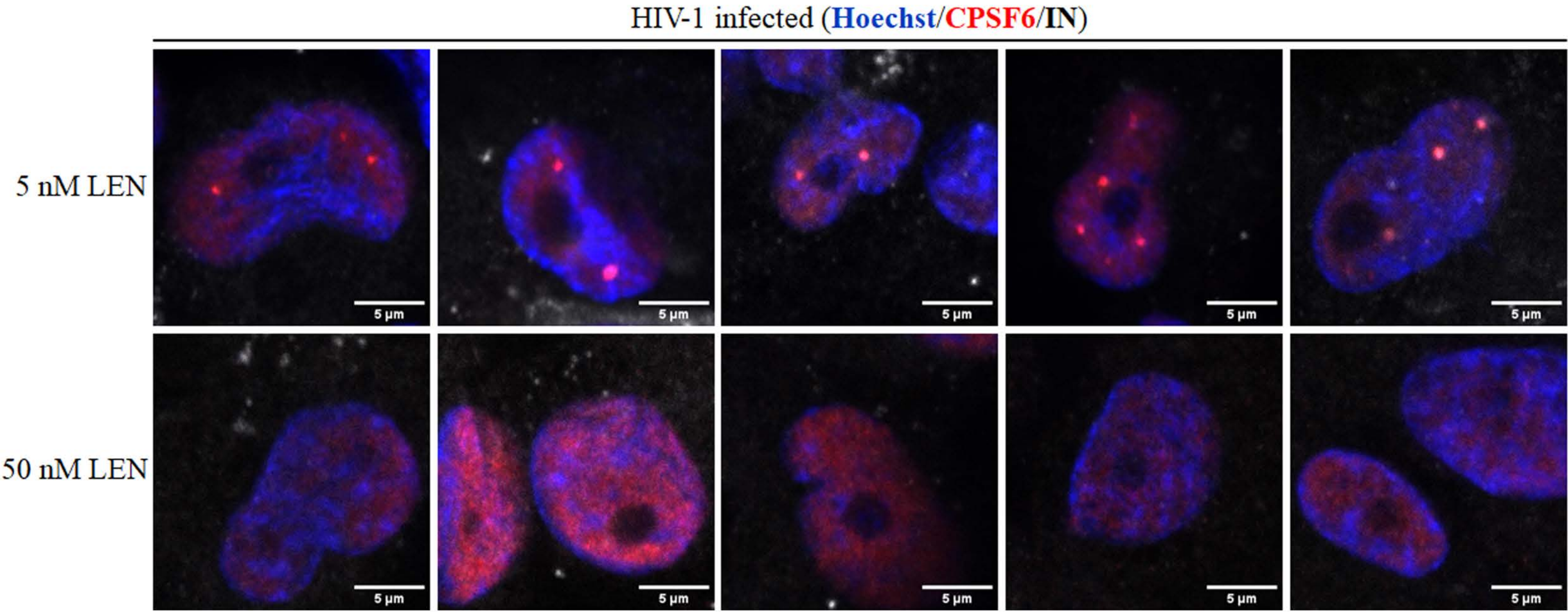

Figure S1

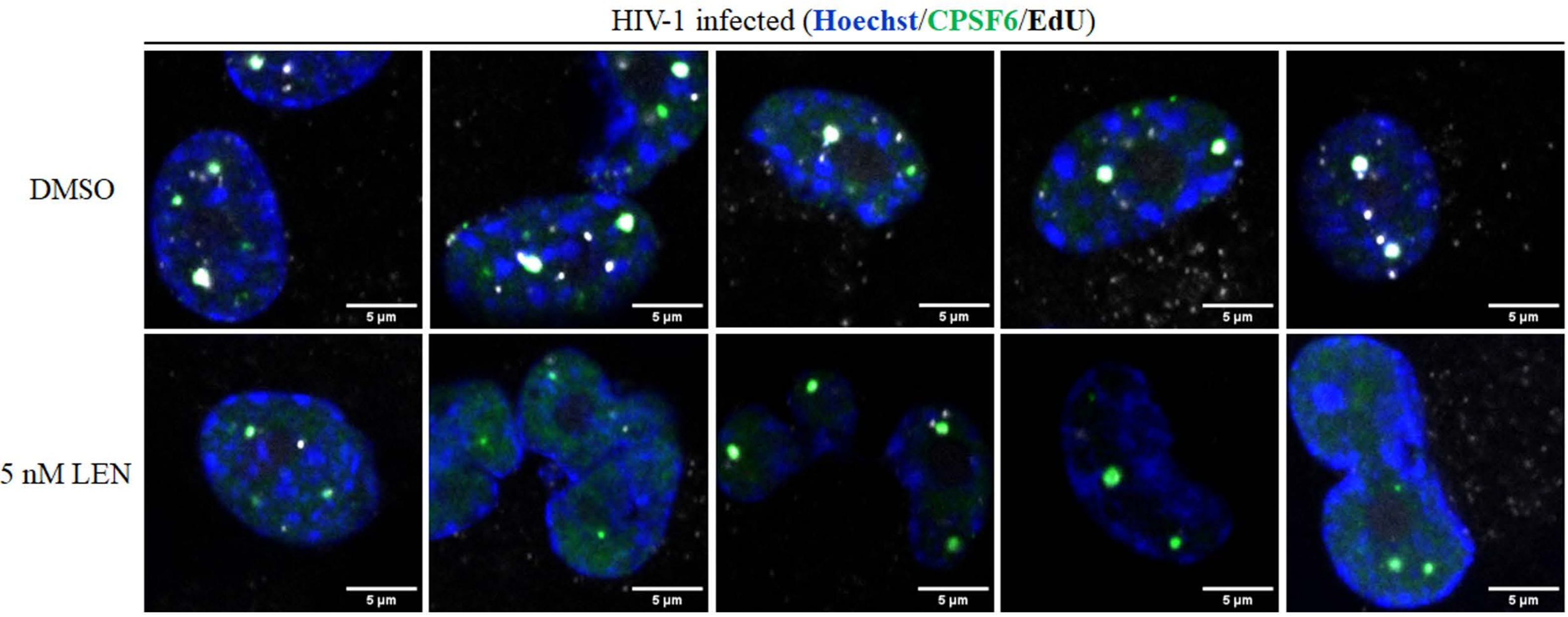

Figure S2

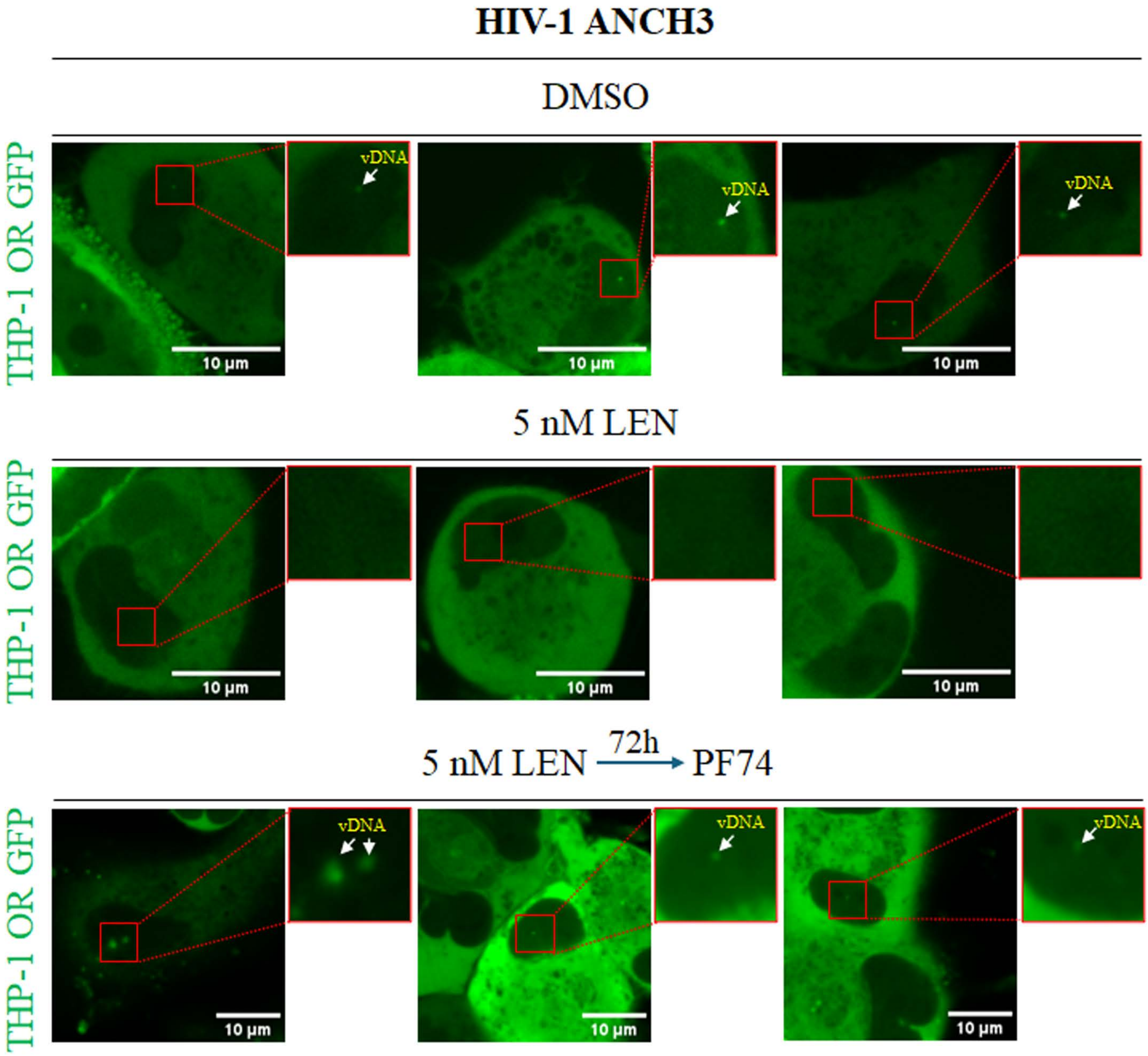

Figure S3

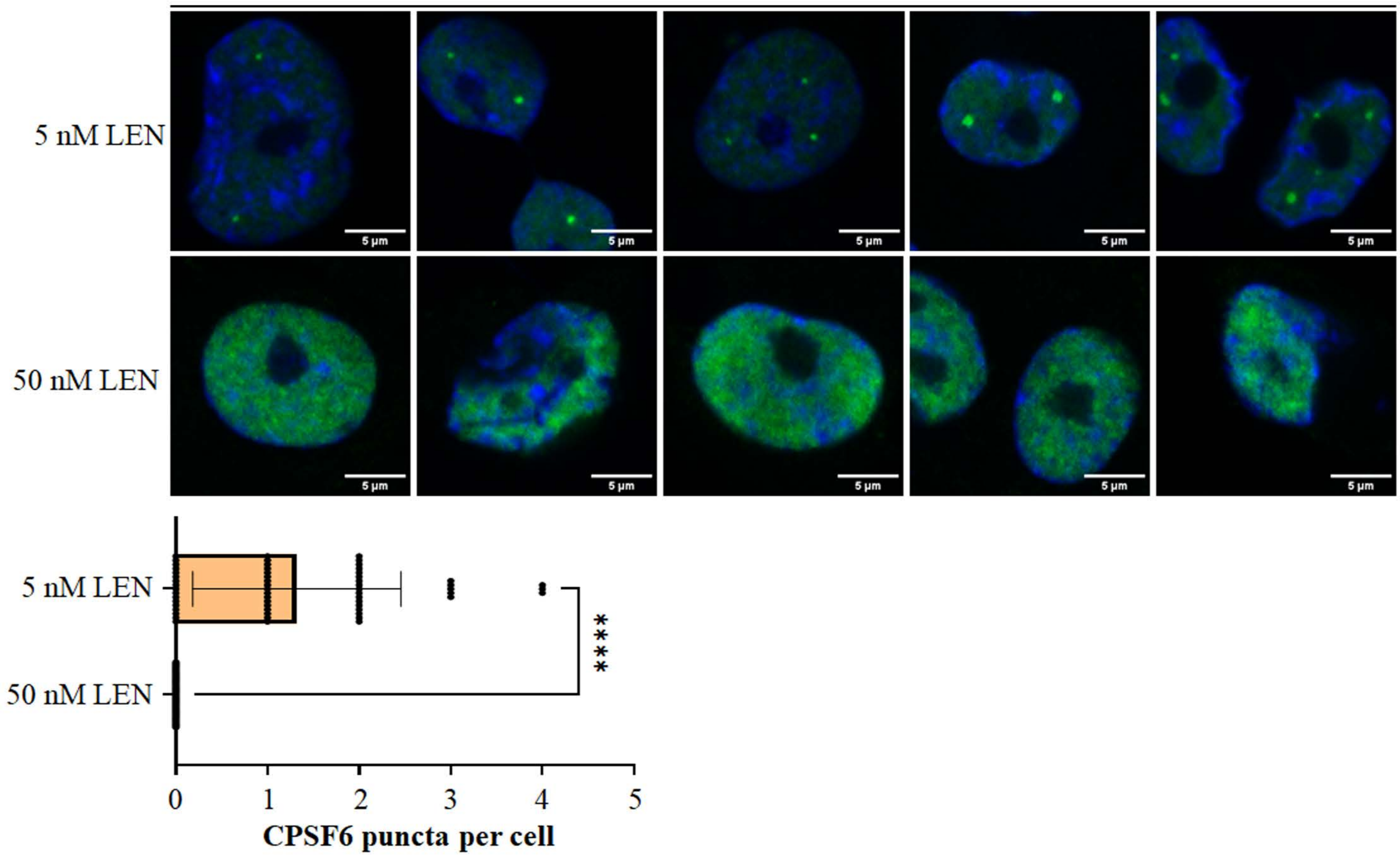
5 nM LEN
50 nM LEN
5 µm
5 nM LEN
50 nM LEN
****
0
1
2
3
4
5
CPSF6 puncta per cell

Figure S4

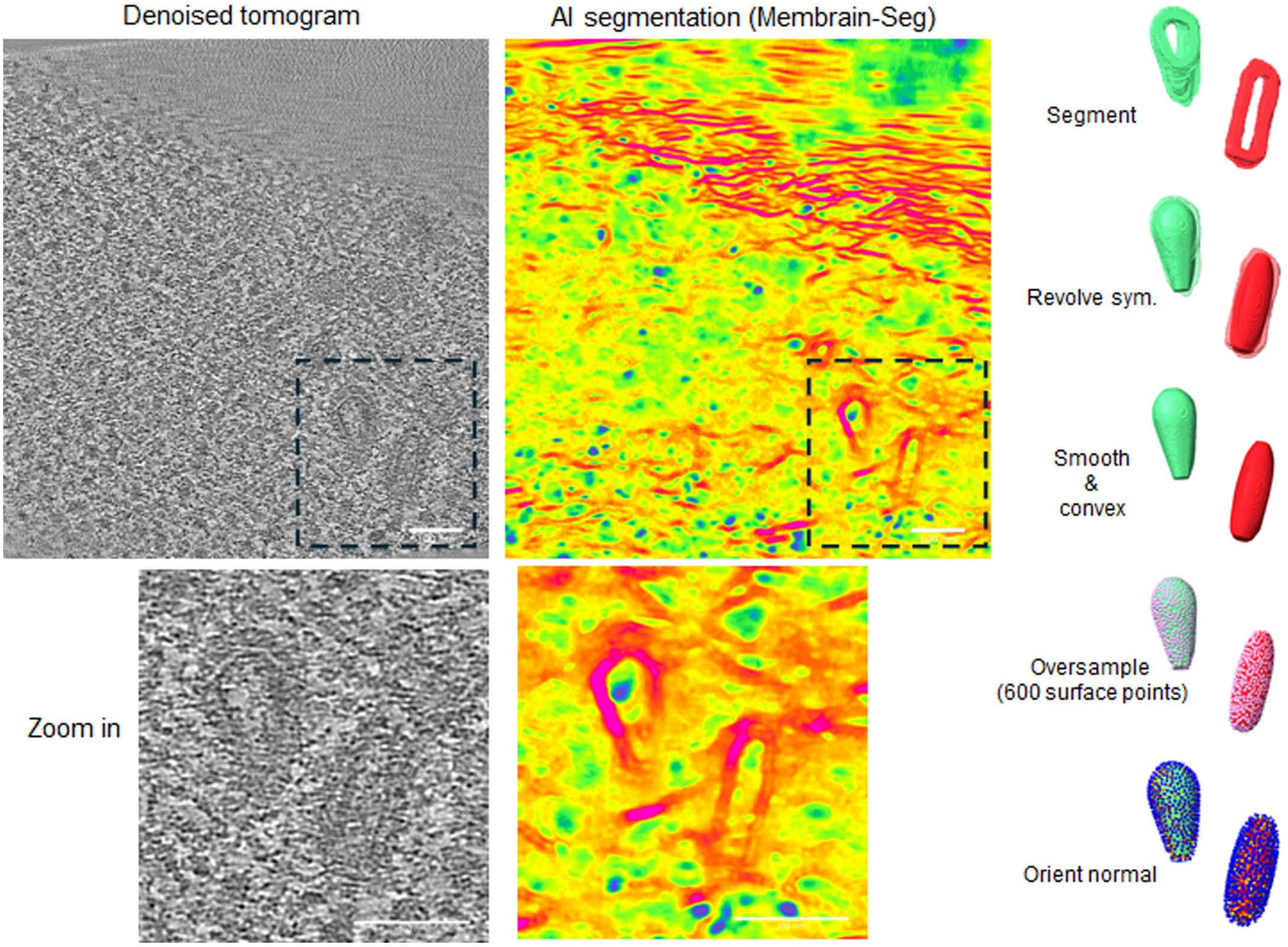
Denoised tomogram
AI segmentation (Membrain-Seg)
Zoom in
Segment
Revolve sym.
Smooth
&
convex
Oversample
(600 surface points)
Orient normal

Figure S5

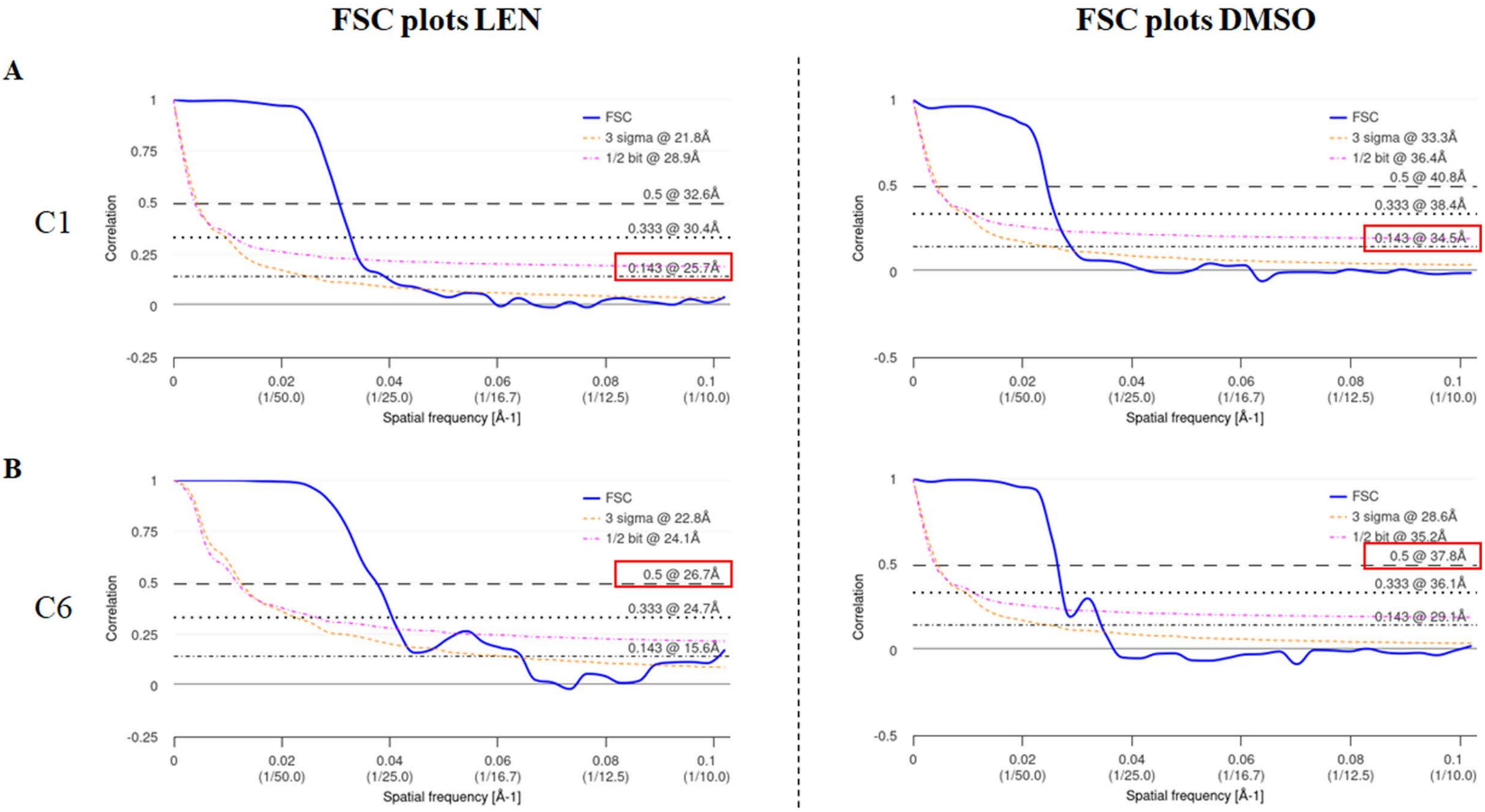

FSC plots LEN
FSC plots DMSO
A
B
C1
C6
FSC
3 sigma @ 21.8Å
1/2 bit @ 28.9Å
0.5 @ 32.6Å
0.333 @ 30.4Å
0.143 @ 25.7Å
3 sigma @ 33.3Å
1/2 bit @ 36.4Å
0.5 @ 40.8Å
0.333 @ 38.4Å
0.143 @ 34.5Å
3 sigma @ 22.8Å
1/2 bit @ 24.1Å
0.5 @ 26.7Å
0.333 @ 24.7Å
0.143 @ 15.6Å
3 sigma @ 28.6Å
1/2 bit @ 35.2Å
0.5 @ 37.8Å
0.333 @ 36.1Å
0.143 @ 29.1Å
Correlation
Spatial frequency [Å-1]
0.02 (1/50.0)
0.04 (1/25.0)
0.06 (1/16.7)
0.08 (1/12.5)
0.1 (1/10.0)

Figure S6

A

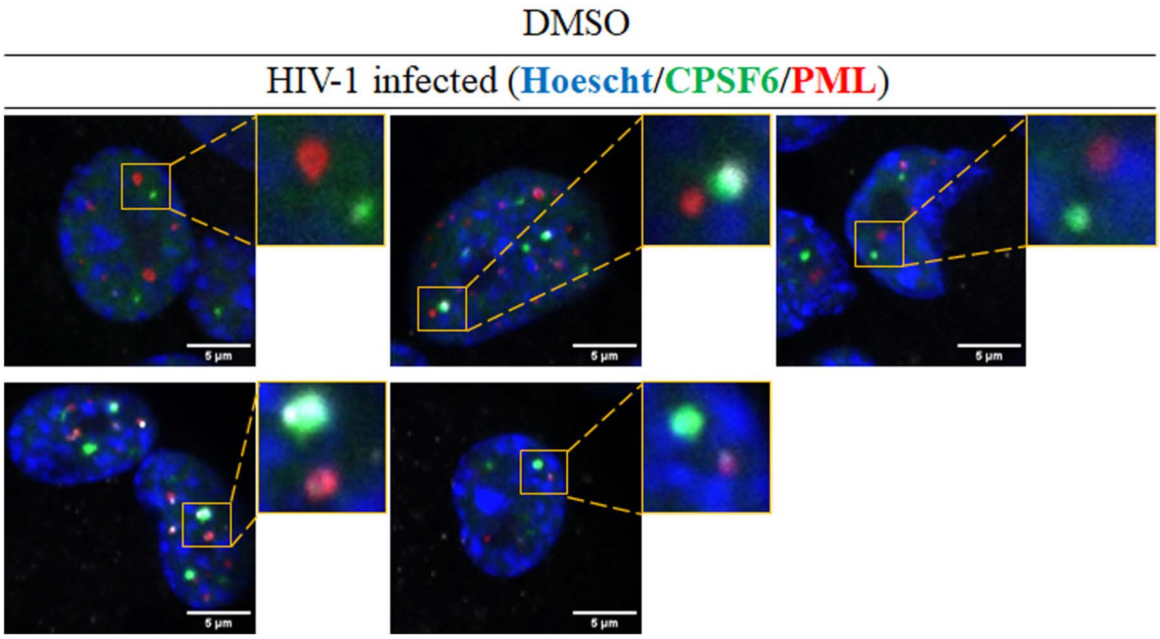
DMSO
HIV-1 infected (Hoescht/CPSF6/PML)

B

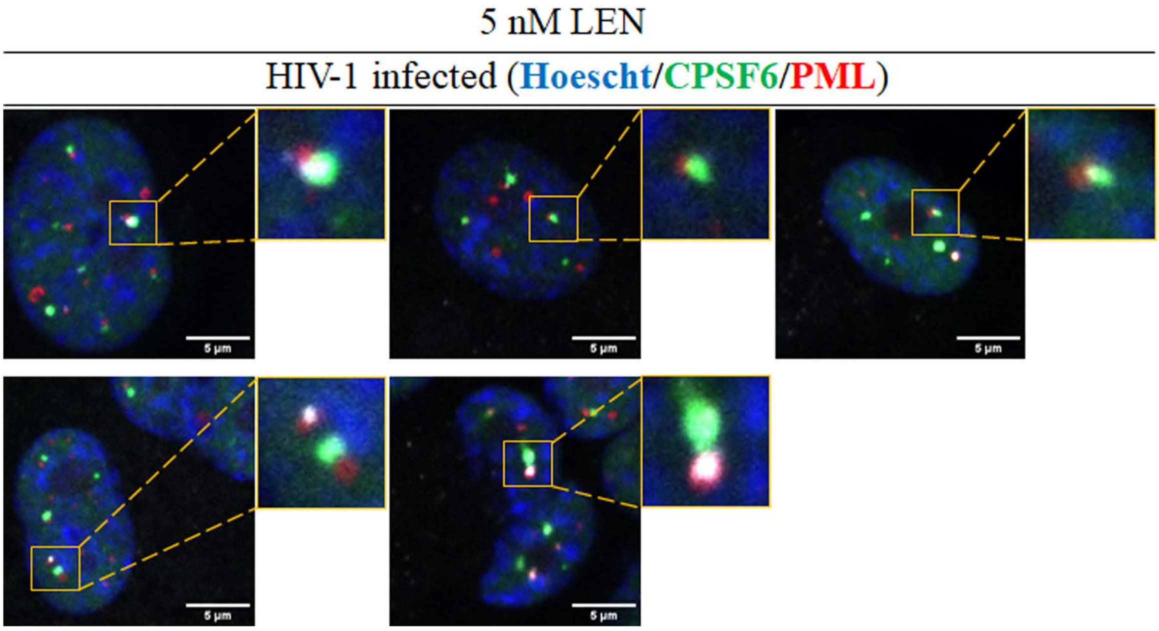
5 nM LEN
HIV-1 infected (Hoescht/CPSF6/PML)

Figure S7

A

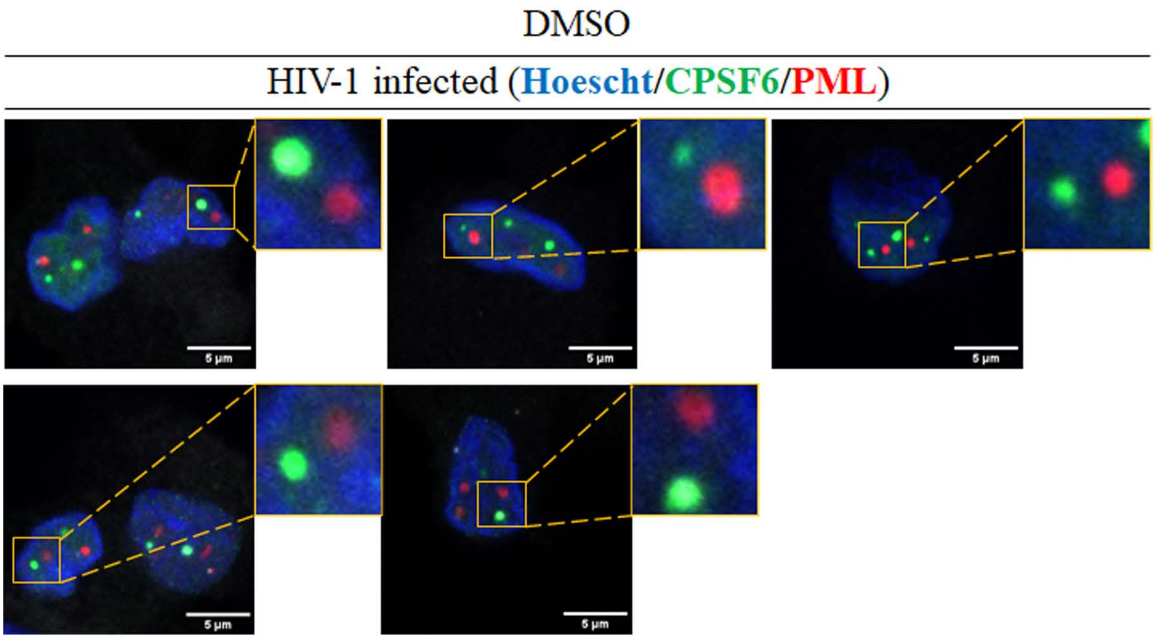
DMSO
HIV-1 infected (Hoescht/CPSF6/PML)
5 µm

B

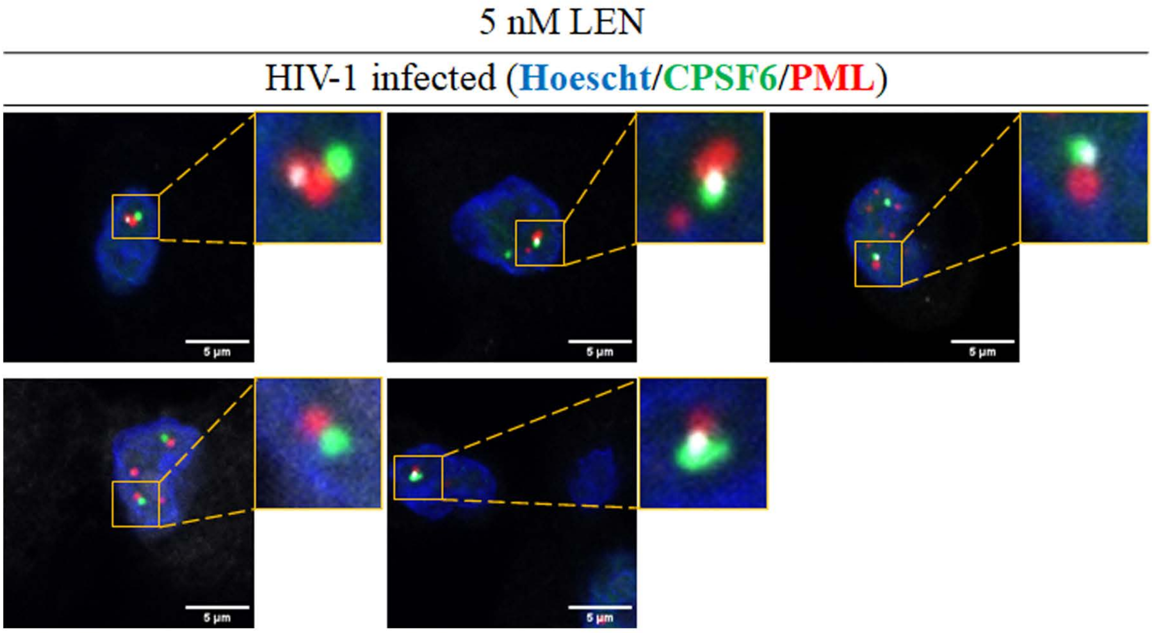
5 nM LEN
HIV-1 infected (Hoescht/CPSF6/PML)
5 µm

Figure S8

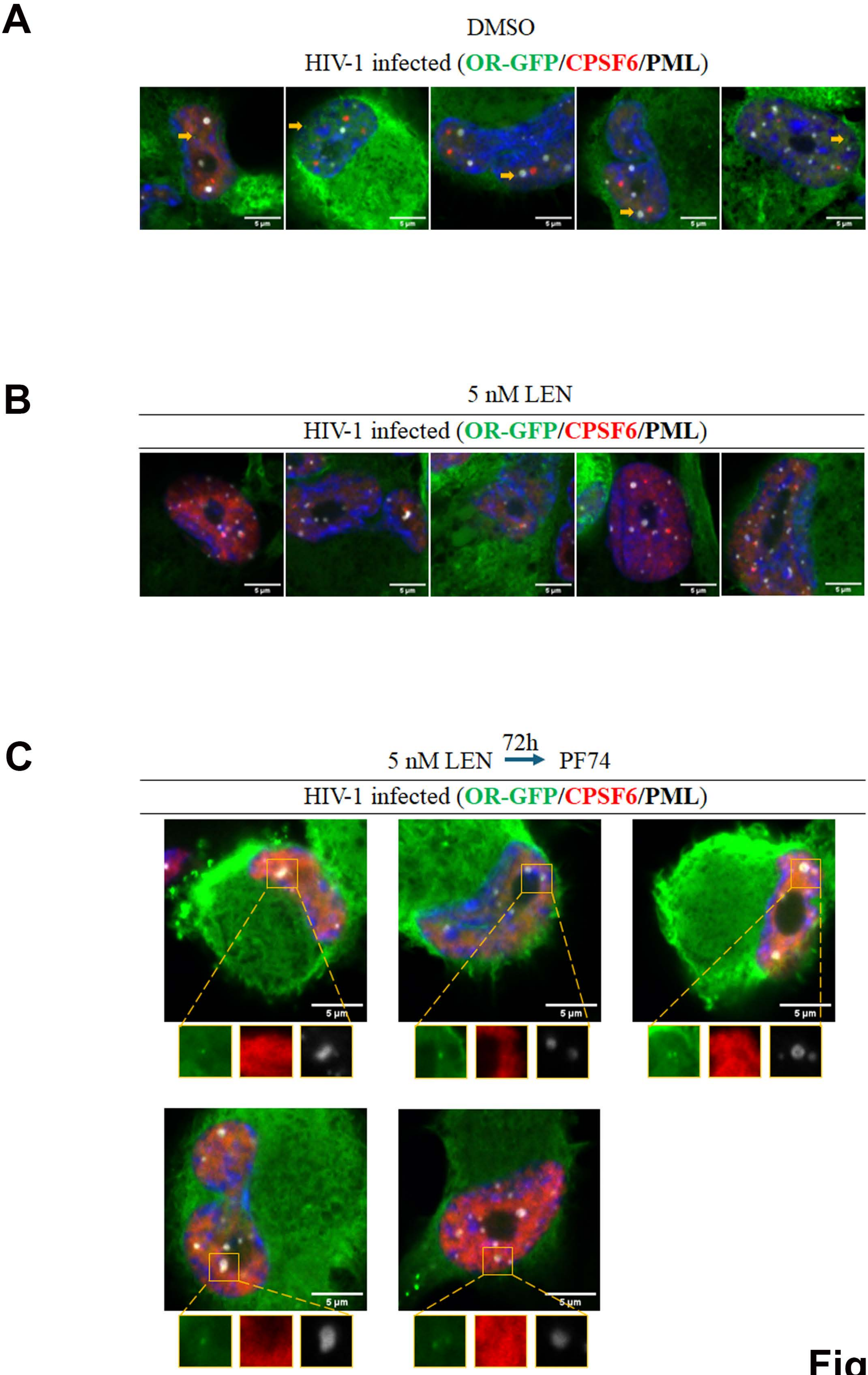
A
DMSO
HIV-1 infected (OR-GFP/CPSF6/PML)
5 µm
B
5 nM LEN
HIV-1 infected (OR-GFP/CPSF6/PML)
C
5 nM LEN
72h
PF74
HIV-1 infected (OR-GFP/CPSF6/PML)

Figure S9

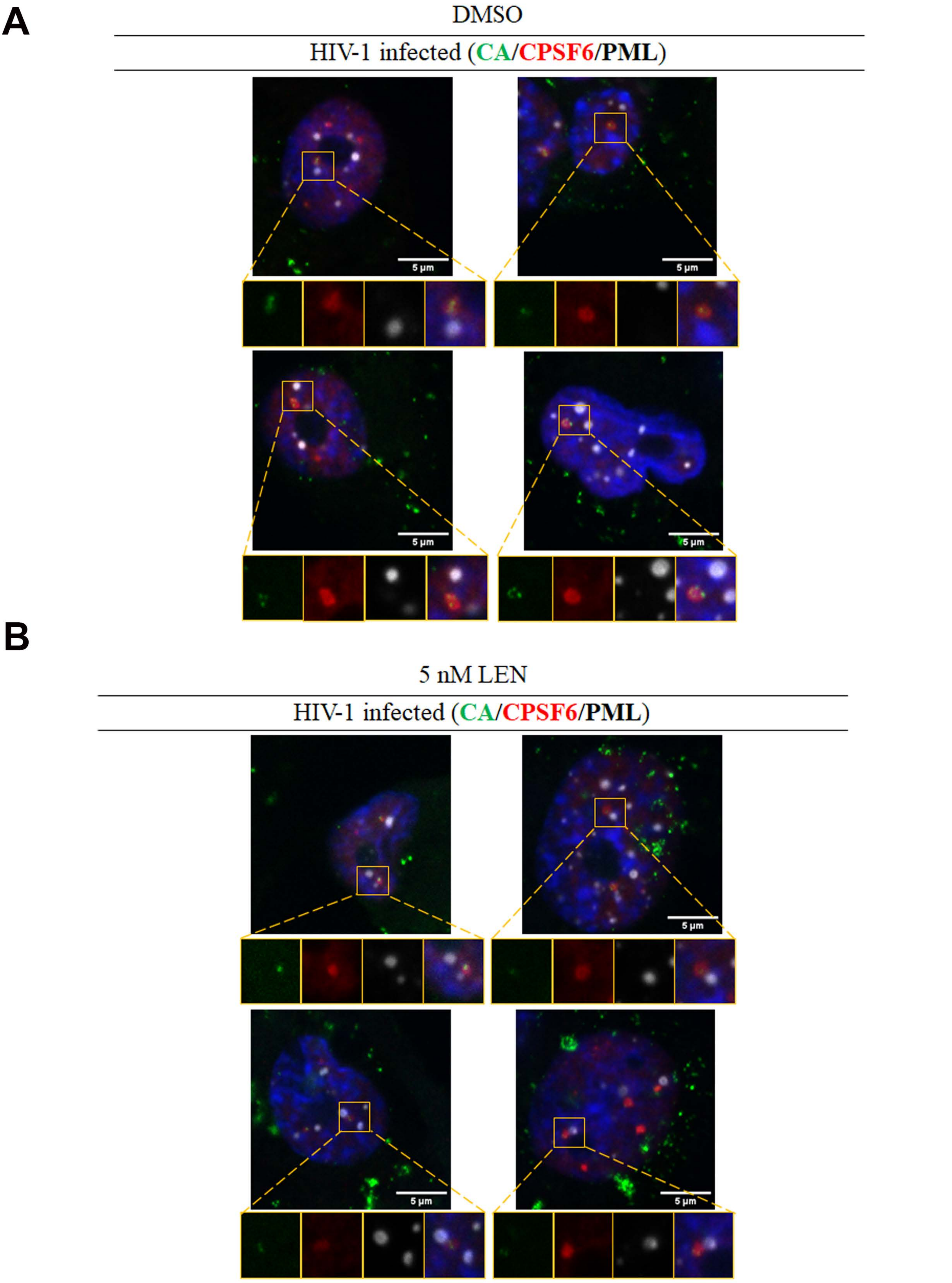
A
DMSO
HIV-1 infected (CA/CPSF6/PML)
5 µm
B
5 nM LEN
HIV-1 infected (CA/CPSF6/PML)
5 µm

Figure S10

Sample	N° unique ISs	intron	exon	inter (out)	#genes (uniques)	#ISs (unique)	#ISs (intron)	#ISs (exon)	#ISs (out)
HIV-1 THP-1	12,641	71.00%	5.00%	24.00%	4,343	12,288	8,957	639	3,044
HIV-1 + 5nM LEN THP-1	3,842	52.02%	3.05%	44.94%	2,113	3,755	1,998	117	1,726

Table S1. Integration sites distribution in introns, in exons, intergeniques.

Table S2. Gene-ontology analysis of HIV-1 RIGs in THP-1 cells after 3 days post-infection

Analysis Type:	PANTHER Overrepresentation Test (Released 20200728)						
Annotation Version and Release Date:	PAN-GO Version 1.0 Released 2022-03-22						
Analyzed List:	HIV1 Over-targeted genes (Homo sapiens)						
Reference List:	Homo sapiens (all genes in database)						
Test Type:	**FISHER**						
Correction:	FDR						
PAN-GO biological process	Homo sapi	upload_1 (	upload_1 (	upload_1 (	upload_1 (	upload_1 (	upload_1 (
regulation of biological process (GO:0050789)	6678	139	91.92	+	1.51	6.98E-09	5.44E-05
protein modification process (GO:0036211)	1312	44	18.06	+	2.44	4.45E-08	1.73E-04
cellular protein modification process (GO:0006464)	1312	44	18.06	+	2.44	4.45E-08	1.15E-04
biological regulation (GO:0065007)	7274	145	100.12	+	1.45	4.62E-08	9.00E-05
regulation of nucleobase-containing compound metabolic process (GO:0019219)	2402	65	33.06	+	1.97	6.92E-08	1.08E-04
regulation of cellular process (GO:0050794)	6341	130	87.28	+	1.49	9.38E-08	1.22E-04
macromolecule modification (GO:0043412)	1438	46	19.79	+	2.32	9.58E-08	1.07E-04
regulation of RNA metabolic process (GO:0051252)	2323	63	31.97	+	1.97	1.20E-07	1.17E-04
regulation of gene expression (GO:0010468)	2649	69	36.46	+	1.89	1.40E-07	1.21E-04
regulation of RNA biosynthetic process (GO:2001141)	2154	59	29.65	+	1.99	2.33E-07	1.81E-04
regulation of transcription, DNA-templated (GO:0006355)	2154	59	29.65	+	1.99	2.33E-07	1.65E-04
regulation of nucleic acid-templated transcription (GO:1903506)	2154	59	29.65	+	1.99	2.33E-07	1.51E-04
regulation of macromolecule metabolic process (GO:0060255)	3168	77	43.61	+	1.77	3.14E-07	1.88E-04
Unclassified (UNCLASSIFIED)	6902	56	95	-	0.59	3.57E-07	1.98E-04
biological_process (GO:0008150)	13949	231	192	+	1.2	3.57E-07	1.85E-04
regulation of nitrogen compound metabolic process (GO:0051171)	2941	72	40.48	+	1.78	8.05E-07	3.92E-04
regulation of macromolecule biosynthetic process (GO:0010556)	2300	60	31.66	+	1.9	9.29E-07	4.25E-04
cellular macromolecule metabolic process (GO:0044260)	2260	59	31.11	+	1.9	1.21E-06	5.24E-04
cellular protein metabolic process (GO:0044267)	1742	49	23.98	+	2.04	1.45E-06	5.95E-04
regulation of primary metabolic process (GO:0080090)	2989	72	41.14	+	1.75	1.50E-06	5.85E-04
regulation of cellular biosynthetic process (GO:0031326)	2322	60	31.96	+	1.88	1.58E-06	5.87E-04
regulation of biosynthetic process (GO:0009889)	2331	60	32.08	+	1.87	1.67E-06	5.91E-04
regulation of metabolic process (GO:0019222)	3369	78	46.37	+	1.68	2.18E-06	7.37E-04
regulation of transcription by RNA polymerase II (GO:0006357)	1788	49	24.61	+	1.99	3.31E-06	1.07E-03
histone modification (GO:0016570)	166	12	2.28	+	5.25	3.40E-06	1.06E-03
regulation of cellular metabolic process (GO:0031323)	3089	72	42.52	+	1.69	4.91E-06	1.47E-03
protein metabolic process (GO:0019538)	1978	52	27.23	+	1.91	5.69E-06	1.64E-03
histone H3-K36 methylation (GO:0010452)	4	3	0.06	+	54.49	1.02E-05	2.84E-03
intracellular signal transduction (GO:0035556)	541	21	7.45	+	2.82	2.14E-05	5.75E-03
macromolecule metabolic process (GO:0043170)	3163	70	43.54	+	1.61	4.21E-05	1.09E-02
peptidyl-lysine modification (GO:0018205)	138	9	1.9	+	4.74	1.27E-04	3.20E-02
peptidyl-lysine dimethylation (GO:0018027)	8	3	0.11	+	27.24	1.37E-04	3.34E-02
histone lysine methylation (GO:0034968)	37	5	0.51	+	9.82	1.45E-04	3.42E-02

Table S3. Oligonucleotides used in this study.

**Name**	**Sequence (5'-3')**
**MH531**	TGTGTGCCCGTCTGTTGTGT
**MH532**	GAGTCCTGCGTCGAGAGAGC
**LRT probe**	(FAM)-CAGTGGCGCCCGAACAGGGA-(TAMRA)
**MH535**	AACTAGGGAACCCACTGCTTAAG
**MH536**	TCCACAGATCAAGGATATCTTGTC
**2LTR probe**	(FAM)-ACACTACTTGAAGCACTCAAG-GCAAGCTTT-(TAMRA)
**Alu166**	TCCCAGCTACTCGGGAGGCTGAGG
**Alu2**	GCCTCCCAAAGTGCTGGGATTACAG
**LAMBDA U5**	ATGCCACGTAAGCGAAACTC TGGCTAACTAGGGAACCCACTG
**AE3013**	ATGCCACGTAAGCGAAACTC
**AE990**	CTGACTAAAAGGGTCTGAGG
**AE995**	(FAM)- TTAAGCCTCAATAAAGCTTGCCTTGAGTGC-(TAMRA)
**MxA-F**	ATCCTGGGATTTTGGGGCTT
**MxA-R**	CCGCTTGTCGCTGGTGTCG
**CXCL10-F**	TGGCATTCAAGGAGTACCTC
**CXCL10-R**	TTGTAGCAATGATCTCAACACG
**Actin For**	AACACCCCAGCCATGTACGT
**Actin Rev**	CGGTGAGGATCTTCATGAGGTAGT
**Actin Probe**	(FAM)-CCAGCCAGGTCCAGACGCAGGA-(BHQ1)

Supplementary methods

Coarse-grained models

Initial structures and direct coexistence simulations: To investigate the coexistence of condensed and dilute phases in CPSF6-based condensates, we performed direct coexistence simulations using a slab geometry (91). This approach simulates both the condensed and diluted phases in an NVT ensemble within the same simulation box, enabling the system to sample equilibrium coexistence densities. Protein structures were initially modeled using AlphaFold3 (Abramson2024), to identify structured domains. Globular domains were defined as high-confidence regions (pIDDT > 90) extending for at least 30 residues in length. Specifically, we assigned the globular domains to residues 81–177 and 411–486 for CPSF6, 55–102 for SRRM2, and 580–855 for PML-1. Regions with lower confidence were treated as fully flexible and modeled as intrinsically disordered regions (IDRs). The coarse-grained representation placed one bead per amino acid centered at the Cα atom, following the Mpipi model parameterization (90). Short initial simulations employing Langevin dynamics were run for each individual chain (80 ns at 300 K) to obtain realistic protein conformations prior to slab formation. For each system, 500 protein chains were positioned on a cubic grid with a minimum spacing of 12 nm to avoid steric overlap. All systems were simulated at near-physiological conditions, namely 300 K and 150 mM monovalent salt, using periodic boundary conditions. The simulations consisted of two sequential stages: (i) Compression stage (NPT ensemble). The system was equilibrated for 200 ns using an isotropic Berendsen barostat at 0.9 atm to compact the system and establish a condensed phase. (ii) Direct coexistence stage (NVT ensemble). The simulation box was subsequently expanded sixfold along the x-axis to create a slab geometry containing both dense and dilute regions. Simulations were then continued for 2 μs under NVT conditions until the densities of both phases reached equilibrium, allowing the formation of a stable liquid–liquid interface. All simulations employed a Langevin Middle integrator with a 10 fs timestep**.** Coordinates were saved every 100 ps in XTC format for post-simulation analysis. To examine how increasing concentrations of viral DNA (vDNA) influence the composition and stability of CPSF6 condensates, we performed a series of direct coexistence simulations at fixed protein concentration and varying vDNA content. vDNA chains are negatively charged polymers composed of 500 DNA base-pair beads. These beads interact with amino acid residues, capturing non-specific nucleic acid–protein interactions. Each titration system contained 500 CPSF6 chains and an increasing number of vDNA molecules, corresponding to base pair-to-amino-acid ratios (0.0, 0.1, 0.2, 0.3, 0.4, and 0.5). To explore the effect of SRRM2, we run a

direct coexistence simulation of 250 copies of SRRM2 together with 250 copies of SRRM2 in the presence of DNA at a 0.1 ratio (DNA base pairs: amino acids). To investigate whether PML-1 can interact with CPSF6 condensates containing vDNA, we performed three-component direct coexistence simulations including CPSF6, PML-1, and vDNA. Each system consisted of 300 CPSF6 and 150 PML-1 chains, along with negatively charged vDNA polymers corresponding to a base-pair-to-amino-acid ratio of 0.1. Initial configurations were generated using the same slab construction protocol described previously.

Density calculations: we quantified phase coexistence by computing one-dimensional density profiles along the slab normal (x-axis) from the last 100 ns of each NVT trajectory. Per frame, atomic coordinates were (i) wrapped under periodic boundary conditions, (ii) recentred so that the protein density peak lay at $x = 0$ using the circular mean of protein x positions, and (iii) histogrammed along x over the interval $[-L_x/2, L_x/2]$ with a bin width of 2 nm. Counts in each bin were divided by the corresponding bin volume, $\Delta V = \Delta x\, L_y\, L_z$, to yield number densities multiplying by per-bead masses and converting to mass densities. Protein and nucleic-acid beads used residue-specific coarse-grain masses consistent with the Mpipi mapping. Framewise profiles were then averaged to obtain the mean and standard deviation for protein, nucleic acid, and total (protein + nucleic acid).

Partition coefficient: the partition coefficient between condensed and dilute phases was computed from the one-dimensional density profiles of vDNA and protein obtained from the coexistence simulations. For each system, the protein density profile was used to define condensed and dilute regions. The condensed phase was operationally defined as all spatial bins with protein density exceeding the median across the simulation box, while the remaining bins were assigned to the dilute phase**.** The total number of beads in each phase was obtained by integrating the corresponding density over each region. The fractional occupancy of nucleic acids in the condensed phase was calculated as:

$$f_{\text{condensed}} = \frac{N_{\text{Particle Type,condensed}}}{N_{\text{Particle Type,total}}},$$

with the complementary fraction in the dilute phase $f_{dilute} = 1 - f_{condensed}$. The average densities of vDNA in each phase were then used to compute the partition coefficient:

$$P = \frac{C_{condensed}}{C_{dilute}},$$

where $C_{condensed}$ and C_{dilute} are the mean vDNA densities in the condensed and dilute regions, respectively.

Standard deviations were propagated according to:

$$\sigma_P = P\sqrt{\left(\frac{\sigma_c}{\mu_c}\right)^2 + \left(\frac{\sigma_d}{\mu_d}\right)^2},$$

Where μ_c and μ_d are the mean densities and σ_c, σ_d are their respective standard deviations within each phase.

To evaluate whether density differences between partition coefficients were statistically significant, two-tailed Welch's t-tests were performed between condensed-phase densities (and, separately, dilute-phase densities) across independent simulation replicates.

Protein sequences used for Coarse-Grained Models

> CPSF6 Human (Uniprot number: Q16630)
MADGVDHIDIYADVGEEFNQEAEYGGHDQIDLYDDVISPSANNGDAPEDRDYMDTLP
PTVGDDVGKGAAPNVVYTYTGKRIALYIGNLTWWTTDEDLTEAVHSLGVNDILEIKF
FENRANGQSKGFALVGVGSEASSKKLMDLLPKRELHGQSPVVTPCNKQFLSQFEMQS
RKTTQSGQMSGEGKAGPPGGGSRAAFPQGGRGRGRFPGAVPGGDRFPGPAGPGGPPP
PFPAGQTPPRPPLGPPGPPGPPGPPPPGQVLPPPLAGPPNRGDRPPPPVLFPGQPFGQPP
LGPLPPGPPPPVPGYGPPPGPPPPQQGPPPPPGPFPPRPPGPLGPPLTLAPPPHLPGPPPG
APPPAPHVNPAFFPPPTNSGMPTSDSRGPPPTDPYGRPPPYDRGDYGPPGREMDTARTP
LSEAEFEEIMNRNRAISSSAISRAVSDASAGDYGSAIETLVTAISLIKQSKVSADDRCKV
LISSLQDCLHGIESKSYGSGSRRERSRERDHSRSREKSRRHKSRSRDRHDDYYRERSRE
RERHRDRDRDRDRERDREREYRHR
>SRRM2 Human (Uniprot number: Q9UQ35)
MYNGIGLPTPRGSGTNGYVQRNLSLVRGRRGERPDYKGEEELRRLEAALVKRPNPDI
LDHERKRRVELRCLELEEMMEEQGYEEQQIQEKVATFRLMLLEKDVNPGGKEETPGQ

RPAVTETHQLAELNEKKNERLRAAFGISDSYVDGSSFDPQRRAREAKQPAPEPPKPYS
LVRESSSSRSPTPKQKKKKKKKDRGRRSESSSPRRERKKSSKKKKHRSESESKKRKHR
SPTPKSKRKSKDKKRKRSRSTTPAPKSRRAHRSTSADSASSSDTSRSRSRSAAAKTHTT
ALAGRSPSPASGRRGEGDAPFSEPGTTSTQRPSSPETATKQPSSPYEDKDKDKKEKSAT
RPSPSPERSSTGPEPPAPTPLLAERHGGSPQPLATTPLSQEPVNPPSEASPTRDRSPPKSP
EKLPQSSSSESSPPSPQPTKVSRHASSSPESPKPAPAPGSHREISSSPTSKNRSHGRAKRD
KSHSHTPSRRMGRSRSPATAKRGRSRSRTPTKRGHSRSRSPQWRRSRSAQRWGRSRSP
QRRGRSRSPQRPGWSRSRNTQRRGRSRSARRGRSHSRSPATRGRSRSRTPARRGRSRS
RTPARRRSRSRTPTRRRSRSRTPARRGRSRSRTPARRRSRTRSPVRRRSRSRSPARRSGR
SRSRTPARRGRSRSRTPARRGRSRSRTPARRSGRSRSRTPARRGRSRSRTPRRGRSRSRS
LVRRGRSHSRTPQRRGRSGSSSERKNKSRTSQRRSRSNSSPEMKKSRISSRRSRSLSSPR
SKAKSRLSLRRSLSGSSPCPKQKSQTPPRRSRSGSSQPKAKSRTPPRRSRSSSSPPPKQK
SKTPSRQSHSSSSPHPKVKSGTPPRQGSITSPQANEQSVTPQRRSCFESSPDPELKSRTPS
RHSCSGSSPPRVKSSTPPRQSPSRSSSPQPKVKAIISPRQRSHSGSSSPSPSRVTSRTTPRR
SRSVSPCSNVESRLLPRYSHSGSSSPDTKVKPETPPRQSHSGSISPYPKVKAQTPPGPSL
SGSKSPCPQEKSKDSLVQSCPGSLSLCAGVKSSTPPGESYFGVSSLQLKGQSQTSPDHR
SDTSSPEVRQSHSESPSLQSKSQTSPKGGRSRSSSPVTELASRSPIRQDRGEFSASPMLK
SGMSPEQSRFQSDSSSYPTVDSNSLLGQSRLETAESKEKMALPPQEDATASPPRQKDK
FSPFPVQDRPESSLVFKDTLRTPPRERSGAGSSPETKEQNSALPTSSQDEELMEVVEKS
EEPAGQILSHLSSELKEMSTSNFESSPEVEERPAVSLTLDQSQSQASLEAVEVPSMASSW
GGPHFSPEHKELSNSPLRENSFGSPLEFRNSGPLGTEMNTGFSSEVKEDLNGPFLNQLE
TDPSLDMKEQSTRSSGHSSSELSPDAVEKAGMSSNQSISSPVLDAVPRTPSRERSSSASS
PEMKDGLPRTPSRRSRSGSSPGLRDGSGTPSRHSLSGSSPGMKDIPRTPSRGRSECDSSP
EPKALPQTPRPRSRSPSSPELNNKCLTPQRERSGSESSVDQKTVARTPLGQRSRSGSSQE
LDVKPSASPQERSESDSSPDSKAKTRTPLRQRSRSGSSPEVDSKSRLSPRRSRSGSSPEV
KDKPRAAPRAQSGSDSSPEPKAPAPRALPRRSRSGSSSKGRGPSPEGSSSTESSPEHPPK
SRTARRGSRSSPEPKTKSRTPPRRRSSRSSPELTRKARLSRRSRSASSSPETRSRTPPRHR
RSPSVSSPEPAEKSRSSRRRRSASSPRTKTTSRRGRSPSPKPRGLQRSRSRSRREKTRTT
RRRDRSGSSQSTSRRRQRSRSRSRVTRRRRGGSGYHSRSPARQESSRTSSRRRRGRSRT
PPTSRKRSRSRTSPAPWKRSRSRASPATHRRSRSRTPLISRRRSRSRTSPVSRRRSRSRTS
VTRRRSRSRASPVSRRRSRSRTPPVTRRRSRSRTPTTRRRSRSRTPPVTRRRSRSRTPPV
TRRRSRSRTSPITRRRSRSRTSPVTRRRSRSRTSPVTRRRSRSRTSPVTRRRSRSRTPPAIR
RRSRSRTPLLPRKRSRSRSPLAIRRRSRSRTPRTARGKRSLTRSPPAIRRRSASGSSSDRS
RSATPPATRNHSGSRTPPVALNSSRMSCFSRPSMSPTPLDRCRSPGMLEPLGSSRTPMSV

LQQAGGSMMDGPGPRIPDHQRTSVPENHAQSRIALALTAISLGTARPPPSMSAAGLAA
RMSQVPAPVPLMSLRTAPAANLASRIPAASAAAMNLASARTPAIPTAVNLADSRTPAA
AAAMNLASPRTAVAPSAVNLADPRTPTAPAVNLAGARTPAALAALSLTGSGTPPTAAN
YPSSSRTPQAPASANLVGPRSAHATAPVNIAGSRTAAALAPASLTSARMAPALSGANLT
SPRVPLSAYERVSGRTSPPLLDRARSRTPPSAPSQSRMTSERAPSPSSRMGQAPSQSLLP
PAQDQPRSPVPSAFSDQSRCLIAQTTPVAGSQSLSSGAVATTTSSAGDHNGMLSVPAPG
VPHSDVGEPPASTGAQQPSALAALQPAKERRSSSSSSSSSSSSSSSSSSSSSSSSGSSSSD
SEGSSLPVQPEVALKRVPSPTPAPKEAVREGRPPEPTPAKRKRRSSSSSSSSSSSSSSSSSS
SSSSSSSSSSSSSSSSSSSSSSSSPSPAKPGPQALPKPASPKKPPPGERRSRSPRKPIDSLRD
SRSLSYSPVERRRPSPQPSPRDQQSSSSERGSRRGQRGDSRSPSHKRRRETPSPRPMRH
RSSRSP

> PML-1 Human (Uniprot number: P29590)

MEPAPARSPRPQQDPARPQEPTMPPPETPSEGRQPSPSPSPTERAPASEEEFQFLRCQQC
QAEAKCPKLLPCLHTLCSGCLEASGMQCPICQAPWPLGADTPALDNVFFESLQRRLS
VYRQIVDAQAVCTRCKESADFWCFECEQLLCAKCFEAHQWFLKHEARPLAELRNQS
VREFLDGTRKTNNIFCSNPNHRTPTLTSIYCRGCSKPLCCSCALLDSSHSELKCDISAEI
QQRQEELDAMTQALQEQDSAFGAVHAQMHAAVGQLGRARAETEELIRERVRQVVA
HVRAQERELLEAVDARYQRDYEEMASRLGRLDAVLQRIRTGSALVQRMKCYASDQE
VLDMHGFLRQALCRLRQEEPQSLQAAVRTDGFDEFKVRLQDLSSCITQGKDAAVSKK
ASPEAASTPRDPIDVDLPEEAERVKAQVQALGLAEAQPMAVVQSVPGAHPVPVYAFSI
KGPSYGEDVSNTTTAQKRKCSQTQCPRKVIKMESEEGKEARLARSSPEQPRPSTSKAV
SPPHLDGPPSPRSPVIGSEVFLPNSNHVASGAGEAEERVVVISSSEDSDAENSSSRELDD
SSSESSDLQLEGPSTLRVLDENLADPQAEDRPLVFFDLKIDNETQKISQLAAVNRESKF
RVVIQPEAFFSIYSKAVSLEVGLQHFLSFLSSMRRPILACYKLWGPGLPNFFRALEDINR
LWEFQEAISGFLAALPLIRERVPGASSFKLKNLAQTYLARNMSERSAMAAVLAMRDL
CRLLEVSPGPQLAQHVYPFSSLQCFASLQPLVQAAVLPRAEARLLALHNVSFMELLSA
HRRDRQGGLKKYSRYLSLQTTTLPPAQPAFNLQALGTYFEGLLEGPALARAEGVSTPL
AGRGLAERASQ

Pipeline for HIV core segmentation and subtomogram averaging.

Two sets of tomogram reconstructions were generated for this study. For denoising and manual segmentation, tilt series binned fourfold (bin 4) were reconstructed in IMOD (bio3d.colorado.edu/imod/, (61) using weighted back-projection (WBP) with a SIRT-like filter applied for 10 iterations. A second reconstruction was generated at bin 2 using WBP with CTF

correction (phase flipping) applied via IMOD. These higher-resolution tomograms (final voxel size approximately 4.9 Å) were used for subtomogram extraction and averaging.

The bin-4 tomograms were denoised using Cryo-CARE, trained for each dataset with even/odd reconstructions following the original noise2noise strategy. The denoised tomograms were used as input for CNN-based segmentation. Segmentation was performed with MemBrain-seg (62). Although the network was trained for membranes, its probability maps also contained enough signal from the thin capsid walls, which have a comparable thickness to membranes of about 5 nm. Instead of applying a fixed probability threshold, the continuous probability maps were examined together with the denoised tomograms during manual segmentation. This allowed low- confidence regions to be identified more easily than with direct thresholding. Manual segmentation was performed slice by slice in the XY plane using Napari (napari.org/). The denoised tomogram and MemBrain probability map were overlaid, and each core was traced across consecutive slices. Accurate tracing was feasible only in the XY plane because along the other orthoslices, the missing-wedge and low contrast made boundaries difficult to define. The top and bottom regions of each core often remained incomplete and were later filled computationally using a local revolve-symmetry procedure. Each segmented object was separated using the connected-components-3d Python package (pypi.org/project/connected-components-3d/). All voxels belonging to each component were converted into Cartesian coordinates, generating a point cloud per segmented core. For each partially segmented core, the main axis was estimated by principal component analysis (PCA) using scikit-learn (scikit-learn.org/). The first principal component defined the approximate long axis of the object and served as a reference for the filling step. To fill the gaps left by manual tracing, an in-house revolve-symmetry algorithm was applied. Along the first PCA axis, the algorithm slices through the object in single-voxel steps. At each position, it calculated the mean radial distance of existing points in that plane. If a plane contained no points, new points were generated at the estimated radius to close the gap. This operation affected only missing sections and did not impose global rotational symmetry on the entire structure. Manual segmentation can introduce small discontinuities between neighboring XY slices, and points added through revolve symmetry can also introduce local discontinuities. These might affect the normal to surface axis estimation of sampled points at a later stage. To reduce these inconsistencies, each point was replaced by the mean position of its neighboring points within a small cubic window (4 voxel cube). This localized smoothing preserved the overall geometry while minimizing slice-to-slice variation introduced during manual tracing.

The filled and smoothed point clouds were converted into convex-hull meshes using Open3D. The convex hull provided a continuous, closed surface suitable for surface sampling. Exactly 600 points were uniformly sampled from each surface (2-3 times oversampled compared to the number of expected hexamers per cone), and vertex normals were calculated using PyMeshLab (https://zenodo.org/records/16314742). These sampled points were used to define the positions and orientations of subtomograms. Each surface was exported in the motivelist format following the conventions used in the TOMtoolbox (63). The motivelists from all tomograms for a single dataset were combined for subtomogram averaging. Subtomograms were extracted from the CTF-corrected bin-2 tomograms using Artiatomi (https://github.com/uermel/Artiatomi). Two initial references were tested. First, the HIV-1 CA hexamer map (EMD-51531, ebi.ac.uk/emdb/EMD-51531/) low-pass filtered to 60 Å using Xmipp tools (64). Second, a featureless, rotationally averaged pentamer reference was used to confirm that the result was not biased by the starting model. Subtomograms were extracted with a box size of 64^3. The first alignment round used a translational search range of ±10 pixels and an angular search of ±25 degrees in 5-degree steps. Alignments were performed with and without imposing C6 symmetry (C1 and C6). At each iteration, particles were ranked by cross-correlation score, and only the top 25% contributed to the updated average to minimize the influence of poorly aligned particles and oversampling. After alignment convergence (6-10 iterations), duplicates within 11 pixels (about 5 nm) were removed, retaining the higher-scoring particle. The remaining particles were inspected manually in ChimeraX (cgl.ucsf.edu/chimerax/) using the ArtiaX plugin (65). Particles outside the expected lattice or with inconsistent orientations were removed. The cleaned particle set was then used to generate a convex hull and resampled again (600 surface points per core) to update the masks and allow a further iteration of alignment. After removing duplicates and all particles that did not organize into a lattice-like distribution, we performed a final round of alignment with gold-standard FSC calculation. Particles were divided into even and odd halves and aligned independently using a reduced search range of ±3 pixels translation and 1 degree rotation within ±5 degrees. The resulting half-maps were compared by Fourier Shell Correlation (FSC) using the EMDB FSC server (ebi.ac.uk/emdb/validation/fsc/), and resolutions were reported at both 0.5 and 0.143 FSC thresholds.

Figure Legends supplementary and movies

Figure S1. 5 nM of LEN allows the formation of HIV-1-MLOs. THP-1 cells were differentiated with PMA (48h) and infected with HIV-1 ΔEnv pseudotyped with VSV-G carrying the HA-tagged IN for 72 hours at a MOI of 20. THP-1 cells were then fixed and stained with both CPSF6 (red) and HA (IN, white) antibodies. The chromatin was stained with the Hoechst 33342 dye (blue). Images of infected cells were recorded by confocal microscopy. The scale bar (white line) is equal to 5 μm.

Figure S2. 5 nM of LEN impairs the release of vDNA from HIV-1 MLOs. After infection of THP-1 cells with HIV-1 (VSV-G ΔEnv IN(HA), BRU) at an MOI=20 for 72 hours, cells were processed for both immunostaining and EdU click-chemistry to stain CPSF6 and vDNA, respectively. Confocal microscopy was used to capture images, showing representative examples (5 per condition) without (upper panel) or with (lower panel) LEN treatment. A white scale bar of 5 μm is included for reference.

Figure S3. Upon LEN treatment (5 nM), the vDNA is not detected by HIV-1 ANCHOR technology. Multiple examples of THP-1 cells stably expressing OR-GFP infected with HIV-1 ANCH3 (MOI=20) for 3 days in the presence or in absence of LEN (5nM). 72 hours after infection some samples were treated with a high dose of PF74 (25 mM). The double-stranded vDNA (OR-GFP dots) is shown in the green channel. Specific nuclear areas are highlighted with red squares. These regions were zoomed in and vDNA foci are indicated with white arrows.

Figure S4. A high dose of LEN (50 nM) dissolves CPSF6 puncta, while a low dose (5 nM) of LEN preserves the clusters intact. **(A)** THP-1 cells were differentiated with PMA (160 nM) and then infected with HIV-1 (VSV-G ΔEnv IN(HA), BRU) at an MOI=10 for 48 hours. Subsequently, the medium was replaced with RPMI supplemented with FBS, antibiotics and LEN (5 and 50 nM). After 24 hours of treatment, cells were fixed, immunostained using a CPSF6 antibody and imaged by confocal microscopy (green channel). The scale bar (white line) represents 5 mm. **(B)** A histogram showing the quantification of clusters per cell treated with either 5 nM LEN or 50 nM LEN for 24 hours (60 cells were analyzed per condition). Data are shown as the mean ± SD. Statistical significance was tested with a Welch's t-test (*****p*-value<0.0001).

Figure S5. Pipeline to segment HIV cones and prepare them for STA. Left: Denoised tomogram of an HIV-infected cell nucleus. The dashed square highlights a region containing two core capsids, one conical and one tubular. Middle: MemBrain-seg probability map for the same region, showing where core-like densities can be detected. These probability maps are overlaid with the denoised tomogram in Napari to guide manual slice-by-slice segmentation. Right: Processing steps applied to each manually segmented core before subtomogram averaging, shown from top to bottom: isolated manual segmentation of each core; gap filling using local revolve symmetry; smoothing of the resulting point cloud; convex-hull surface generation; uniform oversampling of 600 surface points; and assignment of surface normals. These particles are then used for subtomogram averaging and subsequent lattice mapping (see main text Figures 3–4).

Figure S6. Estimation of STA models resolution using Fourier Shell Correlations (FSC) plots. Gold-standard Fourier shell correlation (FSC) plots of subtomogram averaged maps of the HIV-1 capsid (CA) hexamer in presence (left panel) and in absence of LEN (right panel). **(A)** FSC plots of HIV-1 hexamer with red squares highlighting the resolution at 0.143 FSC cut-off for C1 symmetry. **(B)** FSC plots of HIV-1 hexamer with red squares highlighting the resolution at 0.5 FSC cut-off for C6 symmetry. Half maps of each CA hexamer average were independently post-processed on the EMDB-FSC server to generate gold-standard FSC plots.

Figure S7. LEN prompts the vicinity between CPSF6 puncta and PML-NBs with vDNA transfer in THP-1 cells. Multiple images of THP-1 cells infected with HIV-1 ΔEnv pseudotyped with VSV-G (MOI = 20) for 72 hours in presence or in absence of LEN and subsequently processed for immunostaining and EdU-based Click chemistry to visualize viral DNA. Z-stack images were acquired using confocal microscopy with the following fluorescence channels: blue (Hoechst 33342) for nuclei, green for CPSF6, red for PML, and far-red (rendered in white) for EdU-labeled vDNA. **(A)** Confocal images of 5 additional THP-1 cells that were not treated with LEN. **(B)** Confocal images of 5 additional THP-1 cells that were treated with 5 nM LEN. Yellow square indicates the zoom on the images. These are part of the set of experiments shown in figure 6A.

Figure S8. LEN prompts the vicinity between CPSF6 puncta and PML-NBs with vDNA transfer in primary macrophages. Multiple images of primary monocytes from healthy

donors were differentiated into macrophages and infected with HIV-1 for 72 hours in the presence or absence of LEN (5 nM). Shown are confocal images of fixed macrophages immunostained for CPSF6 (green), PML nuclear bodies (red), and viral DNA (white), the latter visualized using EdU Click chemistry. **(A)** Confocal images of 5 additional primary macrophages that were not treated with LEN. **(B)** Confocal images of 5 additional primary macrophages that were treated with 5 nM LEN. Yellow square indicates the zoom on the images. These are part of the set of experiments shown in figure 6C.

Figure S9. The newly synthesized ds vDNA is transferred from CPSF6 puncta to PML-NBs in presence of LEN. Multiple images of THP-1 cells stably expressing OR-GFP were infected with HIV-1 ANCH3 (MOI = 20) for 72 hours. **(A)** Confocal images of fixed cells not treated with LEN. **(B)** Confocal images of cells treated with 5 nM LEN. **(C)** Confocal images of cells treated with 5 nM of LEN before CPSF6 punctadisruption by applying a high concentration of PF74 (25 μM). Confocal imaging was performed following immunostaining with CPSF6 and PML antibodies. The images show OR-GFP (green, ds vDNA), CPSF6 (red, HIV-1–MLOs), and PML (white). The yellow arrows point to the vDNA bound by OR-GFP. Yellow square indicates the zoom on the images. These are part of the set of experiments shown in figure 7A.

Figure S10. LEN promotes the spatial proximity between CPSF6 puncta and PML nuclear bodies without evidence of viral core transfer. Differentiated THP-1 cells were infected with YFP-Vpr–labeled HIV-1 in the presence or absence of LEN. At 3 days post-infection, cells were immunostained for CPSF6 and PML. Confocal microscopy shows HIV-1 cores (green), CPSF6 (red), and PML-NBs (white). **(A)** Confocal images of infected cells without LEN treatment. (B) Confocal images of infected cells treated with 5 nM LEN for 72 hours. Yellow boxes highlight magnified regions. These are part of the set of experiments shown in Fig. 7C.

Movie 1. Visualization of HIV-1 cores within HIV-1 MLOs by cryo-ET upon LEN treatment. Tomographic reconstruction shows HIV-1 cores located within the nucleoplasm of macrophage-like cells treated with 5 nM LEN after 72 hours post-infection. This movie supplements Figure 3.

Movie 2. Cryo-ET imaging of HIV-1 cores within HIV-1 MLOs in the absence of LEN. Tomographic reconstruction shows HIV-1 cores in the nucleus of THP-1 cells 72 hours post-infection without LEN treatment. This movie supplement Figure 3.

Movie 3. **Capsid lattice in presence of LEN.** 3D rotating views displaying recovered ultrastructures of HIV-1 capsids determined via STA in a single tomogram (central slice shown in Fig. 3B, top). Data was obtained within the nucleus of macrophage-like cells in the presence of LEN, 72 hours post-infection. The centroid hexamer obtained via STA (shown in Fig. 4A, left) is placed at the recovered positions and orientations for each segmented capsid. Capsids were reconstructed using two rounds of segmentation and STA (see Methods for details; also see Supplementary for details on the analysis pipeline). The color scale indicates cross-correlation values between the STA average and each retrieved hexamer from the HIV-1 core lattice, ranging from low (red) to high (green). This movie supplements Figures 3-4.

Movie 4. **Capsid lattice in absence of LEN.** 3D rotating views displaying recovered ultrastructures of HIV-1 capsids determined via subtomogram averaging (STA) in a single tomogram (central slice shown in Fig. 3B, bottom). Data was obtained within the nucleus of macrophage-like control cells (without LEN, DMSO), 72 hours post-infection. The centroid hexamer obtained via STA (shown in Fig. 4B, right) is placed at the recovered positions and orientations for each segmented capsid. Capsids were reconstructed using two rounds of segmentation and STA (see Methods for details; also see Supplementary for details on the analysis pipeline). The color scale indicates cross-correlation values between the STA average and each retrieved hexamer from the HIV-1 core lattice, ranging from low (red) to high (green). This movie supplements Figures 3-4.

Movie 5. Pipeline for STA of HIV-1 cores inside the nucleus of macrophage-like cells. Tomographic reconstruction was performed in the Scipion framework, as described in the Pipeline for HIV core segmentation and subtomogram averaging section. The segmentation was performed in the XY plane using the Napari software. The segmented cores were displayed with a range of different colors (from blue to red). 600 points were then uniformly sampled and vertex normals were attributed using PyMeshLab (these points were used to define positions and orientations of subtomograms). The surfaces were exported in the motivelist format according to TOMtoolbox conventions. The subtomograms were subsequently extracted with Artiatomi and used to perform initial STA. The resulting particles with inconsistent positions

or orientations were deleted. The remaining particles were closed with a convex hull and resampled to perform and second round of STA with refined positions. At this stage, particles that did not adopt a lattice-like distribution were removed. The color of particles, for each round of STA, indicates the cross-correlation values between the STA average and each retrieved hexamer from the HIV-1 core lattice, ranging from low (red) to high (green). This movie supplements Figure 4.
